# What Do Patient‐Reported Sleep Measures Assess? A Content Analysis

**DOI:** 10.1111/jsr.70382

**Published:** 2026-06-08

**Authors:** Marie De Bruecker, Elke Veirman, An Mariman, Dirk A. Pevernagie, Katrien B. C. Hertegonne, Fré A. Bauters, Dimitri M. L. Van Ryckeghem, Geert Crombez

**Affiliations:** ^1^ Department of Head and Skin, Faculty of Medicine and Health Sciences Ghent University Gent Belgium; ^2^ Department of Experimental Clinical and Health Psychology, Faculty of Psychology and Educational Sciences Ghent University Gent Belgium; ^3^ Department of Psychiatry and Centre for Integrative Medicine Ghent University Hospital Gent Belgium; ^4^ Department of Internal Medicine and Paediatrics, Faculty of Medicine and Health Sciences Ghent University Gent Belgium; ^5^ Department of Respiratory Medicine AZ Delta Roeselare Belgium; ^6^ Department of Respiratory Medicine Ghent University Hospital Gent Belgium; ^7^ Department of Experimental Health Psychology, Faculty of Psychology and Neuroscience Maastricht University Maastricht the Netherlands

**Keywords:** content analysis, generic sleep questionnaires, international classification of functioning, disability and health, patient‐reported experience measures, patient‐reported outcome measures, review, semantics

## Abstract

Current systematic review analysed the content of generic patient‐reported sleep measures (PRSMs) using (1) the International Classification of Functioning, Disability and Health (ICF) and (2) semantic analysis. A literature search identified 27 PRSMs applicable across multiple sleep disorders. Meaningful concepts within items of these PRSMs were linked to ICF categories using standardised linking rules. Additionally, semantic relations (contextual, causal, temporal or state descriptive) among these concepts were explored. An overview of the ICF content covered within and across questionnaires was compiled. Three main findings emerged. First, the majority of concepts were linked to the ICF component body functions (71.5%), indicating a focus on physiological and psychological symptoms, while activities and participation (14.7%), environmental factors (3.5%), and personal factors (2.2%) were less represented. Second, semantic analysis revealed that multidimensional content often framed symptoms within activities rather than measuring the functional or environmental impact of sleep disorders. Third, the operationalization of similar constructs varied across PRSMs, and their questionnaire labels could diverge from actual item content. This study provides a standardised database source for content‐based evaluation, comparison and selection of PRSMs. Clinicians and researchers should be aware of the conceptual scope of PRSMs to ensure alignment with their specific clinical or research objectives. Recommendations are offered to guide sleep medicine measurement towards a more comprehensive approach integrating biological, psychological, social, environmental and personal aspects.

## 1 Introduction

Sleep disorders are prevalent and may lead to a range of adverse outcomes (Pavlova and Latreille 2019). Since sleep medicine has emerged as a discipline in the 1950s, progress has been made in assessing, diagnosing, and treating patients with sleep disorders (Pevernagie et al. 2025; Shepard et al. 2005). Current management of sleep disorders is strongly guided by the International Classification of Sleep Disorders (ICSD), which categorises sleep disorders into seven areas: insomnia, sleep‐related breathing disorders (SRBD), central hypersomnolence, circadian rhythm sleep–wake disorders (CRSWD), sleep‐related movement disorders (SRMD), parasomnias, and other sleep disorders (American Academy of Sleep Medicine 2023). Diagnosis of these disorders is primarily based on objective markers and certain signs and symptoms that determine the presence of a specific disorder (Pevernagie 2021). Standard diagnostic tools, such as polysomnography (PSG), aim to assess disease severity based on physiological parameters. Cut‐offs for various parameters have been adjusted for sensitivity and specificity (Kapur et al. 2017). A well‐documented example is the apnea‐hypopnea index (AHI), a marker used for diagnosing obstructive sleep apnea (OSA) and for assessing its severity (American Academy of Sleep Medicine Task Force 1999). Although this diagnostic approach has substantially advanced the field, several potential limitations should be considered.

First, there has been a tendency to focus on what is specific or even unique to a disorder, while commonalities across disorders may have been overlooked (Harvey and Sarfan 2024). Research shows that many symptoms of sleep disorders are nonspecific or generic and not tied to a specific nosological entity (Gauld et al. 2021, 2022). Network analyses of ICSD‐3 diagnostic criteria have revealed that many disorders share common symptoms, especially among insomnia disorders, SRBD, and CRSWD (Gauld et al. 2022). Thus, the focus on disorder‐specific features within sleep medicine may inadvertently lead to the underrecognition of generic, yet clinically meaningful symptoms shared across diagnostic categories (Pevernagie 2021). This may result in a fragmented understanding of the patient's experience and the underappreciation of symptoms that fall outside established diagnostic criteria. It also highlights the potential for bias when symptom assessment is confined to a single sleep disorder, as may occur with the use of sleep disorder‐specific questionnaires, which are designed to evaluate outcomes of a particular sleep disorder.

Second, the current approach may create the illusion of homogeneity within each diagnostic category, while considerable heterogeneity is observed even within single diagnostic categories. Recent studies have identified distinct phenotypic profiles within sleep disorders such as insomnia, hypersomnia, and OSA, enabling more homogenous subclassifications based on variables including self‐reported symptoms, comorbidities, and demographic characteristics (Cook et al. 2019; Nyhuis and Fernandez‐Mendoza 2023; Ye et al. 2014; Zinchuk et al. 2017). This heterogeneity suggests that categorical diagnosis may not adequately capture individual variation in clinical presentation.

These challenges highlight the need for a broad multidimensional and transdiagnostic approach to sleep health and sleep disorders. Sleep health is a multidimensional concept, encompassing aspects such as sleep duration, efficiency, timing and sleep‐related daytime impairments (Buysse 2014, 2018). Furthermore, sleep disorders arise from a complex interplay between biological, psychological and social factors and, in turn, can significantly impact these biopsychosocial domains (Verma et al. 2022; Becker et al. 2015; Perrault et al. 2025). A comprehensive assessment should capture symptoms across diagnostic boundaries, acknowledge individual variation, and reflect the multidimensional and biopsychosocial nature of sleep disorders. This underscores the importance of generic sleep patient‐reported outcome measures (PROMs) in delivering patient‐centred care (Ibáñez et al. 2018; Medarov et al. 2013; Pevernagie et al. 2021). These instruments are specifically designed to assess sleep‐related symptoms, aspects of health status and quality of life across a broad spectrum of sleep disorders (Pevernagie et al. 2021). In this paper, we refer to these generic sleep PROMs as patient‐reported sleep measures (PRSMs). Incorporating PRSMs into sleep disorder assessment is increasingly recognised as essential to complement objective diagnostic tools with patient‐reported outcomes (Ibáñez et al. 2018; Medarov et al. 2013; Pevernagie et al. 2021).

Despite the widespread use, healthcare professionals and researchers often face uncertainty about which PROMs to use in clinical practice or research. A challenge lies in identifying and comparing the type of health information that is being collected (Cieza et al. 2016). To allow the analysis of the content of PRSMs, a universal, standardised reference system is key.

The International Classification of Functioning, Disability and Health (ICF) is a framework that comprehensively describes health states from a biopsychosocial perspective (World Health Organization 2001). It describes the individuals' functioning as a dynamic interaction between their health condition, the extent of their functioning and disability, and the influence of contextual factors. A set of linking rules was developed by Cieza et al. to standardise the process of connecting health‐status measures to the ICF framework (Cieza et al. 2016, 2002; Stucki 2005). Several reviews have applied these linking rules to map the content of questionnaires to the ICF framework, including sleep questionnaires (Gradinger et al. 2011; Kaambwa et al. 2024; Noonan et al. 2009; Rat et al. 2008; Sabariego et al. 2015; Sigl et al. 2006; Silva et al. 2021; Stucki et al. 2008). Reviews in this area either focused on sleep disorder‐specific instruments, particularly OSA‐specific questionnaires (Silva et al. 2021; Stucki et al. 2008), or provided only a high‐level overview of ICF content coverage (Gradinger et al. 2011), and were not up‐to‐date with prevailing standards, leaving a gap in our understanding of the constructs and concepts assessed by PRSMs and their interrelations.

Using the ICF as a reference system, this paper investigates whether PRSMs capture the multidimensional nature of sleep disorders. The present work is embedded in a broader investigational approach also covering the scope, structure, and content validity evidence in the development process of PRSMs (Veirman et al. 2026).

Given the transdiagnostic importance of generic PRSMs in the assessment of sleep disorders, the potential for nosological bias associated with disorder‐specific questionnaires, and the absence of a granular content analysis of generic PRSMs, the present systematic review focused exclusively on generic PRSMs.

The current study aimed to (1) conduct an in‐depth ICF content analysis, both on a high level and granular level and (2) compare the content coverage of these PRSMs. However, we observed that the ICF analysis only categorises the different concepts but does not capture the semantic relationships of these concepts within items. For that reason, we conducted an additional exploratory, post hoc semantic analysis.

## 2 Methods

### 2.1 Systematic Review

A systematic review was conducted to identify eligible PRSMs for the use in adults with sleep disorders. This review was reported according to the Preferred Reporting Items for Systematic Reviews and Meta‐analyses (PRISMA) and was preregistered at Open Science Framework (OSF; https://doi.org/10.17605/OSF.IO/NPGK6). The electronic databases Web of Science, PubMed, and PsycINFO were searched in November 2021 to identify eligible studies and questionnaires, with an updated search in December 2025. The terms and Boolean operators specified for the search were (sleep* OR sleep apnea OR insomnia OR restless legs syndrome OR narcolepsy OR parasomnia) for the topic or population of interest, AND (questionnaire* OR scale* OR patient‐reported outcome measure* OR PROM*) AND (psychometric* OR clinimetric*) for the outcome of interest. The search was limited to titles, abstract, keywords, key concepts and table of contents. Additional questionnaires were identified via relevant systematic reviews and meta‐analyses that focus on sleep questionnaires developed for use in adults. A search for other relevant reviews was performed in the grey literature. An additional cited reference search was performed in Web of Science for each included systematic review/meta‐analysis. Other search strategies for the identification of eligible PRSMs were key textbooks and consultation of expert somnologists.

In a first phase, after removing duplicate papers, the titles, abstracts and keywords, and if applicable full texts were screened using the inclusion and exclusion criteria specified in Table 1.

**TABLE 1:** Phase 1: inclusion criteria for studies to identify PRSMs.

Inclusion criteria
English language publications
Human adult participants
Empirical research, study protocols, review papers
Published in peer‐reviewed journals
The study includes one or more self‐report measures

In a second phase, the full texts of the included articles were screened to identify PRSMs that met the inclusion and exclusion criteria outlined in Table 2. Screening was performed by M.D.B. and 10% independently double‐screened by E.V.

**TABLE 2:** Phase 2: inclusion and exclusion criteria for PRSMs.

Inclusion criteria
The questionnaire is a standalone instrument, developed for the use beyond a single study. Single‐item measures are acceptable, but only if they function as standalone instruments.
The questionnaire is developed for use in adults (≥ 18 years).
The questionnaire can be used at both intake and during follow‐up (clinical phase, to assess a patient's sleep disturbances, sleep‐related impairments and health status).
The questionnaire must be validated in patients with (a) sleep disorder(s), not only in healthy participants, either in the development paper or in a follow‐up validation study. This means there are two options: (1) the questionnaire is developed within a population of patients with sleep disorders or (2) the questionnaire is developed in another population (e.g., healthy participants or individuals with other medical conditions) but later validated in a study specifically involving patients with sleep disorders.
The questionnaire can be used in the context of sleep medicine, focusing on sleep disturbances (e.g., sleep initiation or maintenance problems) and/or sleep‐related impairments (e.g., fatigue or sleepiness).
The questionnaire can be unidimensional, addressing one area of sleep disturbance or sleep‐related impairment, or multidimensional, evaluating more than one of these areas. For unidimensional questionnaires specifically, the focus should be on impairments that can be considered direct consequences of poor sleep quality, such as fatigue or sleepiness.
It is a generic sleep questionnaire (i.e., developed for use in multiple sleep disorders or other conditions such as mood disorders or pain syndromes).
Presented in its original form. Some differences in translations, item order, and response scale are accepted. Shortened versions are considered different instruments and should be validated in patients with sleep disorders.

Exclusion criteria
The questionnaire is an other‐report questionnaire, not a self‐report questionnaire.
The questionnaire is a compilation of multiple distinct self‐report measures (i.e., a questionnaire battery) rather than a unified standalone instrument.
The questionnaire is a screening questionnaire, either or not developed in the context of sleep medicine. Screening tools are developed to identify specific sleep disorders, symptoms, or complaints.
The questionnaire is unidimensional and exclusively addresses other daytime impairments, such as anxiety, depression and cognitive impairments. Although these impairments can result from sleep problems, these fall outside the scope and feasibility of the present systematic review.
The questionnaire is treatment‐related. The questionnaire can be used within a sleep medicine context, but it does not specifically address sleep disturbances or sleep‐related impairments. Instead, it is a questionnaire that focuses on aspects related to interventions or treatments, evaluating treatment quality, side effects or motivation to initiate treatment.
The questionnaire is sleep disorder‐specific. The questionnaire is developed for the use in or has a primary focus on particular sleep disorders (i.e., insomnia disorder, restless legs syndrome (RLS), SRBD or OSA, hypersomnia or narcolepsy and parasomnia).

### 2.2 International Classification of Functioning, Disability and Health (ICF) Framework

The ICF framework provides a common language for describing health and health‐related experiences through two main sections (World Health Organization 2001). The first section addresses functioning (positive aspects) and disability (negative aspects) through three components: (1) *body functions* and *structures* include the physiological and psychological systems, as well as the anatomic parts of the human body, together with possible impairments; (2) *activities* capture the execution of tasks and potential limitations; and (3) *participation* denotes involvement in life areas and associated limitations. The second section covers the contextual factors that affect these experiences. *Environmental factors* encompass the physical, social and attitudinal environment in which individuals live and conduct their daily activities, which may act as facilitators or barriers of their functioning. *Personal factors* represent the individual influences on functioning but are not classified in the ICF, such as demographic and lifestyle characteristics.

The ICF uses a hierarchical classification system with over 1450 categories. Each component is subdivided into chapters (level 1) and increasingly detailed categories (up to 4 levels). Categories are identified by unique alphanumeric codes: a letter prefix indicating the component (b for body functions, s for body structures, d for activities and participation, e for environmental factors) followed by up to five digits for specificity.

### 2.3 ICF Linking Rules and Content Mapping

The linking procedure followed established ICF linking rules focusing on content mapping (Cieza et al. 2016, 2002; Stucki 2005). All items, answer options and instructions from the identified PRSMs were compiled in an Excel file for further analysis. Next, concepts within each item were identified. Instructions and response options were included when essential for understanding the item. For example, this was the case for the instructions ‘Please indicate how likely you are to require rest in the following situations …’of the Brugmann Fatigue Scale (BFS) (Mairesse et al. 2019), and the answer options ‘not at all to extremely tired’ of the Fatigue subscale of the Visual Analogue Scale (VAS‐F) (Lee et al. 1991). Within each item, a distinction was made between main (central focus) and additional (supporting information) concepts (Cieza et al. 2016). Additional concepts often provided extra information about the main concepts, as illustrated by the Epworth Sleepiness Scale (ESS) item ‘How likely are you to doze off or fall asleep in the following situations, in contrast to feeling just tired? Sitting, inactive in a public place (e.g., a theatre or a meeting)’, where the main concepts ‘doze off/fall asleep’ and ‘sitting’ and the additional concepts ‘theatre’ and ‘meeting’ were identified (Johns 1991). Each identified concept was linked to the most specific ICF category using ICF Browser, an online application of the ICF classification containing all codes and categories (World Health Organization 2025). Items often contained multiple concepts, which were then each linked separately. For example, ‘I had a hard time concentrating because I was sleepy’ (Patient Reported Outcomes Measurement Information System Item Bank v. 1.0‐Sleep‐related Impairments (PROMIS‐SRI) (Buysse et al. 2010)) contained the concepts ‘hard time concentrating’ and ‘sleepy’ linked to ‘sustaining attention (b1400)’ and ‘energy level (b1300)’ respectively.

In case the information in the item was insufficient to determine the appropriate ICF category, such as ‘I keep going as usual’ (derived from an item of the Sleepiness‐Wakefulness Inability and Fatigue Test (SWIFT) (Sangal 2012)), the concept was designated as ‘not definable’. Information in the items related to general, physical or mental health concepts and general functioning and disability concepts were coded to these categories. In case the information in the item was not captured by any of the ICF categories, such as ‘making mistakes/errors’ (derived from an item of the Sleep Inertia Questionnaire (SIQ)) (Kanady and Harvey 2015), it was attributed ‘not covered’.

### 2.4 Semantic Relations

The ICF coding system lists concepts within items without capturing their relationships. This limitation of the ICF is particularly important for questionnaires including multiple concepts. For example, items of the ESS (Johns 1991), which intend to measure daytime sleepiness, appear to cover activities and participation due to how the concepts within items are mapped using the ICF coding system. For example, the item ‘How likely are you to doze off while sitting and reading?’ measures a body function but also codes to activities and participation. Without considering the relationship between the concepts represented within the item, the ICF coding may be erroneously interpreted as the ESS also measuring reading ability when it actually measures sleepiness in the context of reading.

To address this limitation, we analysed semantic relationships between concepts in selected PRSMs (Johns 1991; Buysse et al. 2010, 1989). To develop a coding scheme, we consulted existing work in the field of semantic relationships (Green et al. 2002), presenting multiple approaches to categorising conceptual relations. Through iterative team discussions, we adapted and refined these approaches for the specific context of sleep health questionnaire items, ultimately identifying four types of semantic relations. First, *cause–effect* relations indicate that one concept (the antecedent) causes an effect (the consequent). For example, in the item ‘How often have you had trouble sleeping (consequent) because you cough or snore loudly (antecedent)?’ of the Pittsburgh Sleep Quality Index (PSQI), trouble sleeping is the consequent of the antecedent coughing or snoring loudly. Second, *contextual* relations assume that a behaviour occurs in certain contexts or situations. In the ESS, the item ‘How likely are you to doze off or fall asleep in the following situations, in contrast to feeling just tired? Sitting and reading.’ illustrates this type of relation. Third, *temporal* relations describe the timing or sequence of behaviours. For instance, the PROMIS‐SRI item ‘I was sleepy during the daytime’. specifies the temporal context of sleepiness. Fourth, *state descriptions* describe a state, sleep‐related or not, without an explicit semantic relation. An example from the Patient Reported Outcomes Measurement Information System Item Bank v. 1.0‐Sleep Disturbance (PROMIS‐SD) is the item ‘My sleep was restful’.

## 3 Results

### 3.1 Selection of PRSMs

An overview of the study and questionnaire selection process is presented in the PRISMA flowchart (Figure 1). Inter‐rater reliability measured by Cohen's Kappa was 0.86, demonstrating strong agreement. The search yielded 8390 records, of which 2901 duplicates were removed, leaving 5489 titles and abstracts for screening. Following full‐text screening of 4158 articles, 2169 studies were identified reporting on sleep questionnaires. From these studies, 107 sleep PROMs were extracted, of which 80 were sleep disorder‐specific and excluded (see Table S1‐Supporting Information), leaving 27 eligible PRSMs that met our inclusion criteria.

**FIGURE 1:**
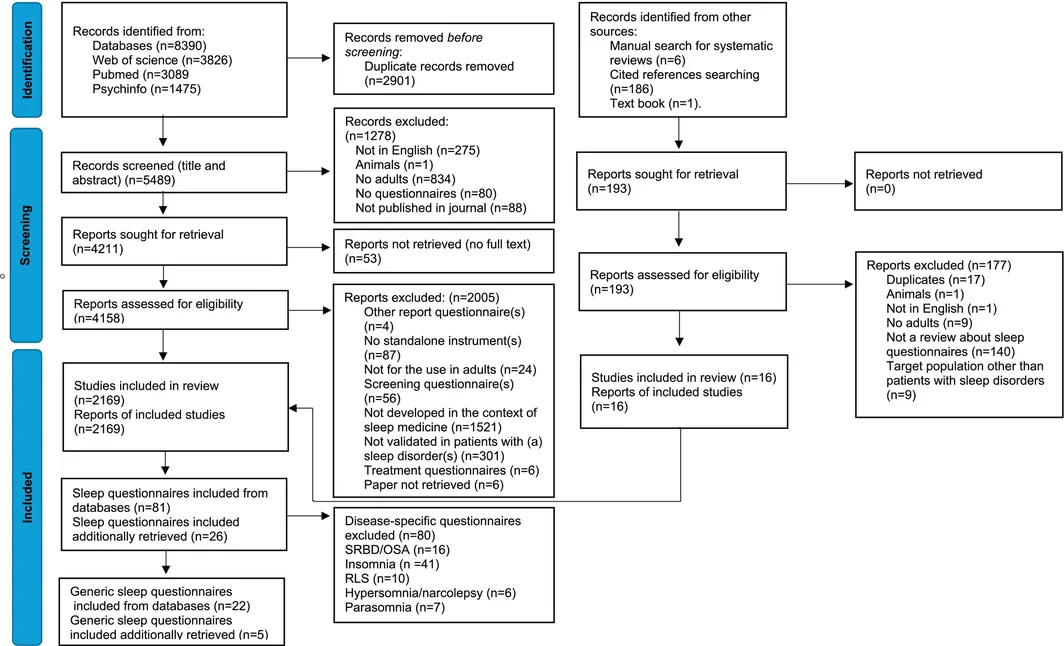
PRISMA flowchart.

### 3.2 Questionnaire Characteristics

Table 3 summarises the 27 included PRSMs with their intended scale content, number of items and response options. Validation populations are detailed in Table S2 (Supporting Information). Most were initially developed and validated in sleep‐disorder populations, typically within hospital sleep units. Four exceptions, that is, the SIQ, Fatigue Severity Scale (FSS), Leeds Sleep Evaluation Questionnaire (LSEQ), and Medical Outcomes Study‐Sleep Scale (MOS‐SS), were not developed in the context of sleep disorders, but were later on validated in patients with sleep disorders. The questionnaires assessed general sleep complaints (*n* = 6), fatigue (*n* = 5), sleepiness (*n* = 5), sleep quality (*n* = 3), combined sleepiness/fatigue (*n* = 3), sleep‐related impairments (*n* = 2), and single questionnaires for unrefreshing sleep, sleep inertia, and sleep timing.

**TABLE 3:** Main characteristics of PRSMs.

#	Questionnaire	Acronym	Intended scale content	Number of items	Answer scale
1	Brugmann Fatigue Scale (Mairesse et al. 2019)	BFS	The need for rest in daily situations	8	4‐point Likert
2	Bologna Questionnaire on Sleepiness‐related Questions (Rinaldi et al. 2001)	BQS	Feelings related to and consequences of excessive daytime sleepiness, sleep habits, snoring and intake of hypnotics	10 main items 12 subitems	Mixed: yes/no, 4‐point Likert
3	Epworth Sleepiness Scale (Johns 1991)	ESS	Propensity of dozing/falling asleep in daily situations	8	4‐point Likert
4	Fatigue, Anergy, Consciousness, Energized and Sleepiness (Shapiro et al. 2002)	FACES	Different experiences related to energy deficit states	50	5‐point Likert
5	Fatigue Severity Scale (Krupp 1989)	FSS	Fatigue severity and its impact	9	7‐point Likert
6	Fatigue subscale of the Visual Analogue Scale (Lee et al. 1991)	VAS‐F	Levels of fatigue and energy	18	Visual analogue scale (VAS)
7	Iowa Sleep Disturbances Inventory (Koffel and Watson 2010)	ISDI	A broad range of sleep complaints	86	Yes/no
8	Leeds Sleep Evaluation Questionnaire (Parrott and Hindmarch 1978)	LSEQ	Impact of hypnotic drugs on aspects of sleep and morning behaviour	10	VAS
9	Medical Outcomes Study‐Sleep Scale (Stewart and Ware 1992; Hays et al. 2005)	MOS‐SS	Different dimensions of sleep	12	Mixed: 5‐and 6‐point Likert, open box
10	Maugeri Sleep Quality and Distress Inventory (Morrone et al. 2017)	MaSQuDI‐17	Impact of sleep disorders on emotional distress	17	3‐point Likert
11	Nonrestorative Sleep Scale (Wilkinson and Shapiro 2013)	NRSS	Unrefreshing sleep	13	Mixed
12	Patient Reported Outcomes Measurement Information System (PROMIS) Item Bank v. 1.0—Sleep Disturbance (Buysse et al. 2010)	PROMIS‐SD	Overall sleep disturbances, quality and satisfaction	27	5‐point Likert
13	Patient Reported Outcomes Measurement Information System (PROMIS) Item Bank v. 1.0—Sleep‐related Impairments (Buysse et al. 2010)	PROMIS‐SRI	Daytime impairments related to sleep or sleep problems	16	5‐point Likert
14	Pittsburgh Sleep Quality Index (Buysse et al. 1989)	PSQI	Sleep quality and sleep disturbances	19	Mixed: 4‐point Likert, open‐ended questions
15	PROMIS Sleep Disturbance Short Form (Yu et al. 2012)	PROMIS‐SD SF	Short form of the PROMIS‐SD	8	5‐point Likert
16	PROMIS Sleep‐related Impairment Short Form 8a (Yu et al. 2012)	PROMIS‐SRI SF	Short form of the PROMIS‐SRI	8	5‐point Likert
17	Resistance to Sleepiness Scale (Violani et al. 2003)	RSS	Likelihood of falling asleep in different situations	12	4‐point Likert scale
18	Revised version of the Medical Outcomes Study Sleep Scale (Yarlas et al. 2021)	MOS Sleep‐R	Revision of MOS‐SS; simplified responses, two recall versions	12	Mixed: 5‐ and 6‐point Likert, open box
19	Sleep Inertia Questionnaire (Kanady and Harvey 2015)	SIQ	Cognitive, behavioural, physiological, and emotional correlates of sleep inertia	22	Mixed: 5‐point Likert scale and open box
20	Sleep Quality Scale (Yi et al. 2006)	SQS	Comprehensive assessment of sleep quality	28	4‐point likert
21	Sleep Timing Questionnaire (Monk et al. 2003)	STQ	Habitual sleep timing	18	Mixed: timing (am/pm), minutes, hours
22	Sleepiness‐wakefulness Inability and Fatigue Test (Sangal 2012)	SWIFT	Difficulty staying awake and fatigue in different situations	12	4‐point Likert
23	Sleep–Wake Activity Inventory (Rosenthal et al. 1993)	SWAI	Multidimensional assessment of EDS	59	9‐point Likert
24	Time of Day Sleepiness Scale (Dolan et al. 2009)	ToDSS	Sleep propensity at different times of the day	8	4‐point Likert
25	Toronto Hospital Alertness Test (Shapiro et al. 2006)	THAT	Perceived alertness	10	6‐point Likert
26	Visual Analog Scale for Tiredness/Sleepiness (Schneider et al. 2004)	VAS‐T/S	Degree of tiredness or sleepiness	1	VAS
27	ZOGIM‐A Alertness Scale (Shapiro et al. 2006)	ZOGIM‐A	The impact of activities on alertness and the expected benefits of increased alertness	10	5‐point Likert

### 3.3 Description of Selected PRSMs: Established and State‐Of‐The‐Art Instruments

Four PRSMs were selected to illustrate ICF and semantic analysis more in depth. This was based on their frequent use in clinical practice and research, and the state‐of‐the‐art manner in which they were developed (Kapur et al. 2017; Johns 1991; Buysse et al. 2010, 1989; Kendzerska et al. 2014; Schutte‐Rodin et al. 2008; Mollayeva et al. 2016).

The ESS is the most commonly used, self‐administered questionnaire for assessing daytime sleepiness in clinical and research settings (Johns 1991; Kendzerska et al. 2014). It evaluates sleep propensity across eight situations on a 4‐point Likert scale with no specified recall period. The total score ranges from 0 to 24, with scores above 10 indicative of EDS (Johns 2000). Given its low cost, ease of administration, simple scoring, and availability in many languages, the ESS is widely and frequently used.

The PSQI is a widely used instrument initially designed to assess sleep quality and sleep disturbances in psychiatric practices and research, and was later adopted in various fields including sleep medicine (Buysse et al. 1989). The PSQI comprises 19 self‐rated items covering seven components: subjective sleep quality, sleep latency, sleep duration, habitual sleep efficiency, sleep disturbances, use of sleeping medication and daytime dysfunction, and 5 bedpartner‐reported items intended for clinical use. Respondents rate their sleep quality and disturbances over the past month using open‐ended questions and a four‐point Likert scale. Component scores (0–3) are summed to yield a global PSQI score (0–21), with a cut‐off score of 5 to discriminate between good and poor sleepers and higher scores indicating greater sleep difficulties.

More recently, the PROMIS initiative developed and validated generic tools that measure general aspects of sleep and sleep‐related impairments in a state‐of‐the‐art manner (Buysse et al. 2010). The PROMIS‐SD aims to measure a broad spectrum of sleep disturbances, including quality and satisfaction, using 27 items. The PROMIS‐SRI evaluates daytime impairments associated with sleep problems through 16 items. Both scales capture these aspects over a one‐week period and utilise a 5‐point Likert scale. Additionally, short forms of the item banks have been derived based on the best‐performing items (Yu et al. 2012).

### 3.4 Results of ICF Linking Procedure Across PRSMs

The number of items across all questionnaires totalled 523 items, from which 908 main concepts and 63 additional concepts were identified. The main concepts were linked to 915 ICF codes. The percentage of main concepts that was linked to the ICF component*s* was as follows: body functions (71.5%), activities and participation (14.7%), environmental factors (3.5%) and personal factors (2.2%). A smaller portion of the concepts could not be defined within the ICF (4.3%) or were associated with general health (0.2%), physical health (0.2%), functioning (0.5%) and disability in general (0.1%). Finally, the smallest portion of the concepts was coded as not covered by the ICF (2.7%) of which a minimal portion was related to health conditions (0.1%).

In total, 134 ICF categories were used to code the content of all PRSMs (see Table S3 ‐Supporting Information). In Table 4, the most frequently mapped categories are outlined, in combination with the number of questionnaires in which they occurred. The category energy level was the most commonly addressed within the body function component, appearing in 21 of 27 questionnaires. This category encompassed concepts such as fatigue, sleepiness, drowsiness, exhaustion, and feeling rested. Among the component activities and participation, the most prevalent category was maintaining a sitting position, occurring in 5 out of 27 questionnaires. Of note, this referred to feeling drowsy or dozing off while sitting in various situations. The environmental factor drugs, referring to sleeping medication, was the most frequently covered. Though included in only three questionnaires, it is an important concept in the LSEQ, appearing in 7 out of its 10 items.

**TABLE 4:** Most frequently mapped categories.

Component	Category	Number of times a concept was linked to the category	Number of PRSMs including the category
Body functions	Energy level	147	21
	Onset of sleep	108	15
	Quality of sleep	58	12
	Maintenance of sleep	52	12
	Functions involving the sleep cycle	47	12
Activities and participation	Maintaining a sitting position	18	5
	Remunerative employment, unspecified	9	6
	Carrying out daily routine, unspecified	8	6
	Socialising	6	5
	Reading	6	5
Environmental factors	Drugs	9	3
	Immediate family	6	1
	General products and technology for communication	5	5
	Attitudes, unspecified	4	2

### 3.5 Results of ICF Linking Procedure for Each PRSM

#### 3.5.1 Biopsychosocial Content

Figure 2 presents the percentage with which ICF components are represented in each PRSM. A complete overview of the linked ICF categories of all generic sleep questionnaires can be found in Table S4 in Supporting Information.

**FIGURE 2:**
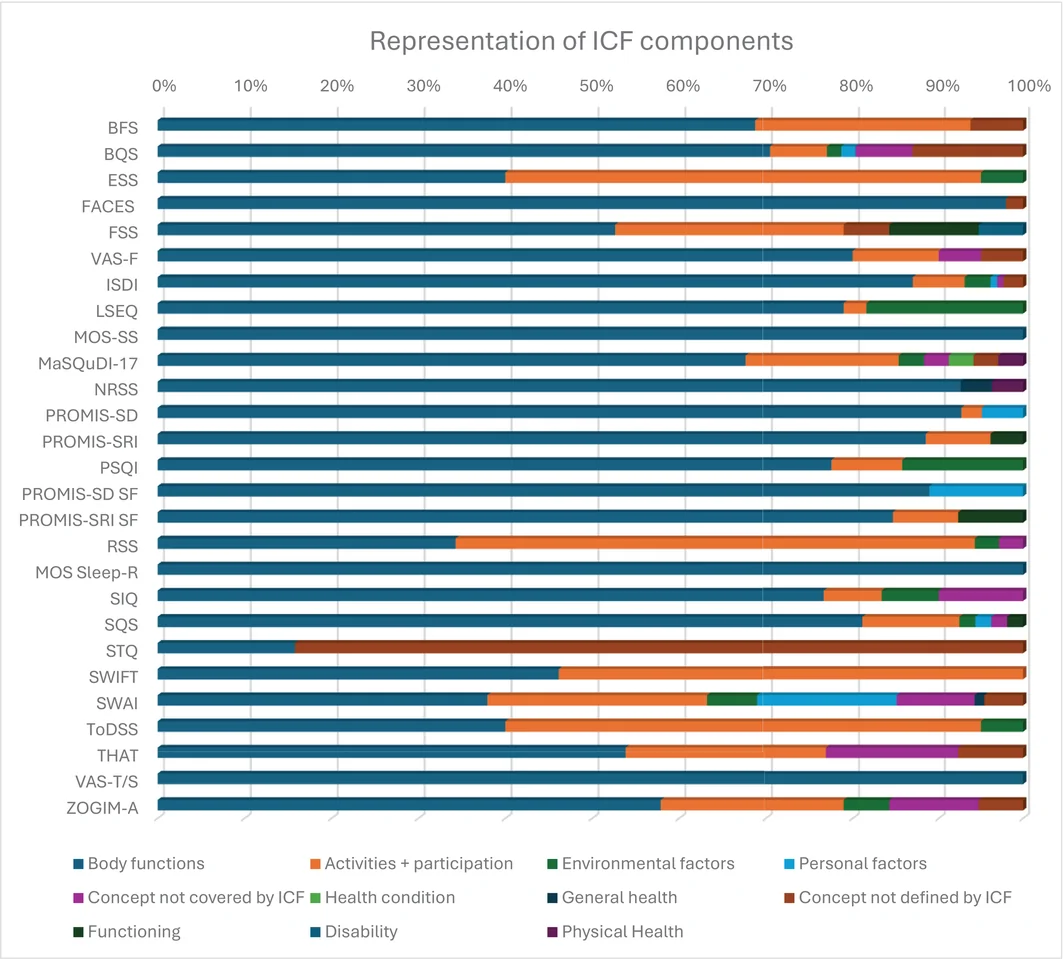
Representation of each ICF component in every PRSM.

In the majority of questionnaires, body functions was the predominantly represented component (81%), with the Fatigue, Anergy, Consciousness, Energized and Sleepiness (FACES), MOS‐SS, the revised version of the Medical Outcomes Study Sleep Scale (MOS Sleep‐R) and the Visual Analog Scale for Tiredness/Sleepiness (VAS‐T/S) questionnaires showing the narrowest scope, focusing only on a subset of body functions. A stronger emphasis on activities and participation was found in four questionnaires, that is, the ESS, Resistance to Sleepiness Scale (RSS), SWIFT and Toronto ssHospital Alertness Test (THAT). Two questionnaires substantially included environmental factors: the PSQI and LSEQ. None of the PRSMs covered the component body structures. Twelve questionnaires included concepts spanning three components: body functions, activities and participation, and environmental factors components (see Figure 2 and Table S4). No clear shift in biopsychosocial content could be observed over time. Of the seven questionnaires developed before 2000, four (LSEQ, PSQI, ESS, SWAI) already incorporated items related to these three components. Among the 20 questionnaires developed after 2000, eight (Bologna Questionnaire on Sleepiness‐related Questions [BQS], RSS, Sleep Timing Questionnaire [STQ], ZOGIM‐A Alertness Scale [ZOGIM‐A], Sleep Quality Scale [SQS], Time of Day Sleepiness Scale [ToDSS], SIQ, and Maugeri Sleep Quality and Distress Inventory [MaSQuDI]) also contained items covering three components. Two of the more recently developed PRSMs (SIQ, MaSQuDI) displayed a slightly broader distribution across ICF components, and the most recently developed PRSM, the BFS, included various activities and participation categories in its additional concepts, reflecting situations in which the need for rest is required. Nevertheless, environmental factors remain underrepresented in both older and newer PRSMs.

Overall, our content analysis showed that questionnaire items could be systematically linked to a large and diverse set of ICF categories. Several questionnaires queried overlapping information, mainly in the sleep functions, energy and drive functions, and emotional functions categories. Conversely, many questionnaires contained unique information, with 57 of the 134 ICF categories identified in only a single questionnaire.

#### 3.5.2 Discrepancy Intended Scale Content and ICF Content

Few recent PRSMs have adopted a multidimensional approach, with only six PRSMs assessing general sleep complaints and one assessing sleep‐related impairments according to their intended scale content (Buysse et al. 2010; Koffel and Watson 2010; Parrott and Hindmarch 1978; Stewart and Ware 1992; Hays et al. 2005; Yu et al. 2012; Yarlas et al. 2021). However, our ICF content analysis revealed that some PRSMs with a seemingly narrow focus, such as the SQS that is ostensibly focused on sleep quality, also captured broader aspects, including influencing sleep factors (e.g., pain in head and neck [b28010]) and sleep‐related impairments (e.g., remunerative employment [d8509]). When compared to a general sleep evaluation PROM like the MOS‐SS, the SQS encompassed more ICF categories in a broader range of chapters. In analysing questionnaires measuring sleepiness (ESS, RSS, SWIFT, ToDSS, BQS and VAS‐T/S), we observed both similarities and differences in how this construct was operationalized. The ESS, RSS and the ToDSS defined it as the likelihood of falling asleep, whereas the SWIFT operationalized it as difficulty staying awake. The BQS distinguished between resistible sleepiness, irresistible sleepiness and sleep attacks, and the VAS‐T/S assessed sleepiness along a continuum from extreme tiredness to full wakefulness. Despite these differences, all PRSMs except the VAS‐T/S, measured sleepiness in the context of activities and participation, with each including items related to reading and the use of transportation. The ESS focused on sedentary situations, while the other questionnaires included a broader spectrum of daily activities across multiple participation domains (e.g., professional, cultural, communication). When comparing the ICF content of two questionnaires assessing sleep quality (the PSQI and SQS), we observed that, aside from limited overlap in core sleep functions and pain, many unique categories were queried per questionnaire. The PSQI targets sensory, respiratory, genitourinary and movement‐related functions that influence sleep, and involves environmental factors such as having a bedpartner and medication use. In contrast, the SQS includes cognitive, emotional and functional domains such as memory and affective difficulties, and work‐related participation.

#### 3.5.3 Biopsychosocial Content of Most Frequently Used PRSMs

Table 5 provides an overview of the linked ICF categories in the four selected PRSMs (Johns 1991; Buysse et al. 2010, 1989). The questionnaires were predominantly linked to categories of body functions and activities and participation, while environmental factors were only minimally covered. The ESS is designed to evaluate the likelihood of falling asleep in various situations. This was reflected in its most frequently endorsed category, onset of sleep. The ESS was primarily linked to activities and participation, as it focuses on dozing off during different activities. The goal of the PSQI is to evaluate overall sleep quality and disturbances. It covered all sleep functions and included sleep‐disturbing factors such as respiratory issues and sleep‐related movement impairments (body functions). Additionally, two PSQI questions addressed sleep‐related impairments in activities and participation. Environmental factors were considered by assessing the impact of the individual's sleep on their roommate or bedpartner. The PROMIS‐SD aims to evaluate sleep disturbances, quality and satisfaction. It included sleep functions, particularly issues related to falling asleep, such as emotional, muscular, and movement‐related disturbances (body functions). However, it did not cover aspects related to the total amount of sleep. The PROMIS‐SRI aims to assess sleep‐related impairments during daytime. Covered impairments included fatigue, sleepiness, and difficulties with cognitive and emotional functioning (body functions) and overall daytime functioning (activities and participation).

**TABLE 5:** ICF content of selected PRSMs.

Chapter	Category	ESS	PSQI	PROMIS‐SD	PROMIS‐SRI
b1	Continuity of consciousness				1
	Orientation functions, unspecified		1		
	Energy level		1	1	8
	Motivation		1		
	Amount of sleep		1	1	
	Onset of sleep	8	2	12	
	Maintenance of sleep		6	4	1
	Quality of sleep		11	7	5
	Functions involving the sleep cycle		1	1	4
	Sleep functions, other specified		2	1	
	Sustaining attention				2
	Regulation of emotion				1
	Range of emotion			2	1
	Emotional functions, other specified			3	
	Control of thought			1	
	Sleep functions, unspecified		1	3	
b2	Sensitivity to temperature		2		
	Sensation of pain, other specified and unspecified		1		
b4	Respiratory rhythm		1		
	Respiration functions, unspecified		1		
	Additional respiratory functions		1		
	Functions of coughing		1		
b6	Frequency of urination		1		
b7	Muscle tone functions, unspecified			1	
	Involuntary contractions of muscles		2		
	Movement functions, other specified and unspecified			1	
d1	Watching	1			
	Reading	1			
d2	Carrying out daily routine, unspecified		1		2
d3	Conversing with one person	1			
	*Conversing with many people*	*1 (A)*			
d4	Lying down	1			
	Maintaining a sitting position	4			
	Using private motorised transportation	1			
	Driving motorised vehicles	1			
	Driving, unspecified		1		
d5	Eating	1	1		
	Ensuring one's physical comfort			1	
d9	Socialising		1		
	*Arts and culture*	*1 (A)*			
e1	Drugs		1		
	General products and technology for communication	1			
e3	Immediate family		6		
Personal factors				2	
Nd‐func					1

*Note:* b1 = mental functions; b2 = sensory functions and pain; b4 = functions of the cardiovascular, haematological, immunological and respiratory systems; b6 = genitourinary and reproductive functions; b7 = neuromusculoskeletal and movement‐related functions; d1 = learning and applying knowledge; d2 = general tasks and demands; d3 = communication; d4 = mobility; d5 = self‐care; d9 = community, social and civic life; e1 = products and technology; e3 = support and relationships; Nd‐func: not definable–functioning; A = additional concept.

In summary, most questionnaires covered various concepts within the ICF classification system. Our analysis also revealed discrepancies between the questionnaires' intended focus and their actual content. Concepts were primarily linked to body functions, yet only four PROMs measured solely this component and there were also PROMs that mainly measured activities and participation. One should be careful in interpreting these findings as only concepts within items were coded separately, but not the relationship between concepts.

#### 3.5.4 Semantic Relations Analyses

In the ESS, all semantic relations between concepts were contextual, indicating that sleepiness was consistently assessed in particular situations. Based on the ICF analysis, the ESS appeared to measure activities and participation (55%) and, to a lesser extent, body functions (40%). However, when accounting for the relationship between concepts within items, it became clear that the ESS primarily assessed sleepiness (body function) during various activity‐related situations (activities and participation). In the PSQI, a mix of semantic relations was identified, including 11 cause–effect relations. According to the ICF, these items assessed sleep quality together with another category such as frequency of urination. When considering the semantic relationship, sleep quality consistently appeared as the consequent of an influencing antecedent factor. Five contextual relationships were identified: one querying sleepiness (body function) during activities, and four in which it was asked whether a bed partner (environmental factor) had observed certain sleep behaviours during the individual's sleep. Other relationships were five state descriptions and three temporal relations. The PROMIS‐SD mainly comprised items with sleep‐related state descriptions, five temporal relations and one cause–effect relation. The ICF analysis indicated that the questionnaire mainly captured sleep functions, and when considering semantic analysis results, the PROMIS‐SD focused on describing the state of sleep itself rather than on contextual or causal factors. The PROMIS‐SRI included seven cause–effect relations, with poor sleep or sleepiness as an antecedent of diverse daytime problems as the consequence, alongside four temporal and five state descriptions. According to the ICF analysis, the PROMIS‐SRI frequently measured sleepiness and sleep functions. The semantic pattern revealed that these body functions are typically framed as antecedents influencing daytime functioning, rather than as outcomes.

In summary, the semantic analysis clarified that many items combine concepts in various relations and revealed which concepts are truly measured as outcomes or rather as context or antecedents within a relationship.

## 4 Discussion

This study analysed the content of 27 PRSMs using the ICF framework and semantic analysis. Three key findings emerged. First, ICF analysis showed that PRSMs predominantly assessed body functions (symptoms and signs) with little coverage of activities and participation and minimal coverage of environmental and personal factors, maintaining a biomedical focus despite the recognised biopsychosocial nature of sleep disorders (Engel 1977). Second, semantic analysis revealed that apparently multidimensional content often merely contextualized symptoms within activities rather than measuring functional outcomes or environmental influences. Third, similar constructs were operationalized differently across PRSMs, and their label or primary focus could diverge from the actual item content. These findings demonstrate that current PRSMs remain narrowly biomedical and fail to provide a comprehensive assessment for understanding sleep disorders as complex biopsychosocial phenomena. We provide a standardised database to support better instrument selection and comparison.

Our finding that generic sleep questionnaires predominantly assessed body functions while neglecting personal and environmental factors confirms previous ICF‐based reviews (Gradinger et al. 2011; Silva et al. 2021), revealing a persistent pattern in the field. Despite decades of recognising sleep disorders as complex phenomena influenced by psychological, social, and environmental factors (Grandner 2017; Szentkirályi et al. 2009), sleep assessment questionnaires have not evolved accordingly. For instance, the MOS‐SS, developed in 1992, dedicates items to different sleep functions and influencing factors such as snoring and headache, classified as body functions (Stewart and Ware 1992; Hays et al. 2005). The instrument does not assess how work schedules affect sleep timing, how sleep deprivation impacts daily activities, or how bedroom environment impacts sleep quality. Importantly, newer questionnaires like the NRSS (2012) show similar distributions, focusing predominantly on body functions such as sleep functions, energy and drive functions and bodily sensations such as pain, rather than incorporating contextual or psychosocial influences (Wilkinson and Shapiro 2013). This pattern suggests that, despite the medical field's shift towards a relational care model that values patient context and participation, this evolution has not yet been reflected in PRSMs.

Second, while the ICF framework revealed a biomedical dominance in PRSMs, it has inherent limitations, which our exploratory, semantic analysis helped to address. The ICF linking rules map each concept in isolation, potentially overestimating multidimensional content. Our semantic analysis uncovered that questionnaires appearing to measure activities and participation often merely use these domains as context for symptom assessment. Taking the PSQI as an example, its item ‘During the past month, how often have you had trouble staying awake while driving, eating meals, or engaging in social activity?’ is linked to both a body function and activities and participation in ICF terms (Buysse et al. 1989). However, semantic analysis revealed this item actually measures sleepiness (the symptom) contextualized within activities, not the actual ability to drive safely, complete meals, or engage socially. This semantic pattern, that is, whereby symptoms were contextualized in activities rather than true functional assessment, revealed that even instruments with seemingly comprehensive ICF coverage maintained a fundamentally symptom‐centric approach. Conversely, in the PROMIS‐SRI, where activities and participation were measured to a lesser extent, semantic relations revealed that these daytime impairments were measured purely here, as a consequence of sleep problems (e.g., ‘In the past 7 days, I had a hard time getting things done because I was sleepy’) (Buysse et al. 2010). The ICF alone cannot capture these nuanced relationships between concepts, a limitation that was previously noted (Gradinger et al. 2011; Rosa et al. 2021), highlighting why our semantic analysis may prove a way forward in understanding what sleep questionnaires truly measure.

Third, the variability in how similar constructs were operationalized across instruments revealed fundamental measurement challenges in sleep assessment. Our analysis furthermore demonstrated clear examples of the jingle fallacy (Peters and Crutzen 2024), that is, although two questionnaires may appear to assess the same construct, they may in fact capture distinct dimensions of these constructs. Instruments labelled as measuring ‘sleep quality’ ranged from the PSQI's focus on physiological and the influence of sleeping behaviours on a bedpartner to the SQS's focus on cognitive, emotional, and functional dimensions (Buysse et al. 1989; Yi et al. 2006). Furthermore, our results showed that reliance on the label or primary content of a questionnaire is insufficient for determining its conceptual scope. We found that some ostensibly unidimensional PRSMs actually capture a broader range of sleep‐related aspects. This misalignment between labels and content means clinicians cannot rely on instrument names when selecting tools, potentially leading to incomplete assessment if chosen based on titles alone. The standardised database developed in this study addresses this challenge by mapping actual content rather than relying on stated purposes.

While the ICF offers a standardised taxonomy for describing health and functioning, it also entails limitations as a reference framework. First, certain ICF categories lack granularity for sleep research and assessment. For example, fatigue, drowsiness, and sleepiness are all linked to the category energy level, and different emotional states are grouped under range of emotion. Second, some concepts could not be defined or covered by the ICF, limiting interpretation when content is considered solely from this framework. Moreover, the model does not yet provide a classification for personal factors; nevertheless, these factors are shown to have an impact on sleep disorders (Grandner 2017; Agarwal 2021; Jehan et al. 2018).

While PRSMs are valuable for assessing sleep disturbances and related impairments, their use requires careful consideration. First, one must critically and systematically evaluate the content of a questionnaire before selecting one. If the goal is to capture particular aspects of the sleep experience, a domain‐specific PRSM can be used (e.g., rest propensity as assessed by the BFS (Mairesse et al. 2019)). However, when aiming to capture the patient's overall health experience, focusing on a single or only few sleep aspects will only provide a partial view. The use of narrowly focused sleep questionnaires in clinical practice may result in a fragmented view of patients with complex sleep problems. To address this limitation, sleep clinics frequently administer multiple questionnaires to capture a comprehensive symptom profile, which can lead to inefficiency and the querying of overlapping information. Recent work of Gauld et al. (2023) highlights this item content overlap in sleep questionnaires. Second, one should be aware that most PRSMs are predominantly symptom‐focused, whereby the insight into the patient's daytime functioning and environment, and how these bidirectionally interact with sleep remains limited. Additional questions about everyday activities and contextual influences should be incorporated during intake consultations. Selection of PROMs should be aligned with study objectives. If the goal is to measure one self‐related symptom, a single‐symptom focused questionnaire might suffice. If the goal is to have a broad evaluation of sleep and its impact, a questionnaire including activities and participation, environmental and personal factor should be prioritised. In addition, including semantic relations in questionnaire analysis provides understanding of how constructs are measured. For example, cause–effect relationships can help disentangle the potential origin of a patient's symptom. However, when a questionnaire only includes cause–effect items, this may also introduce bias in the interpretation of complaints, as it assumes that the symptoms originate from a specific source such as sleep.

We argue that a shift in sleep medicine assessment might be needed: moving from a one size‐fits‐all symptom checklist towards multidimensional sleep assessment integrating symptoms, the impact on daytime functioning, and the influence of environmental and personal factors. Calls for multidimensional PROMs reflect the complexity of sleep experiences: biomedical, psychological, social, and contextual (Verma et al. 2022; Becker et al. 2015; Perrault et al. 2025). Yet, there may be reasons why such questionnaires have not been developed in the past. Extensive questionnaires can be lengthy and burdensome, which may not be feasible in routine clinical practice or suitable for all purposes. Therefore, a modular approach may offer a better solution, using a core questionnaire for essential constructs and optional modules tailored to specific objectives or purposes. This enhances flexibility, reduces redundancy, and improves efficiency while staying relevant to the patient's experience. One example is illustrated within The PROMIS framework (Cella et al. 2007), another example is the current development of the European Sleep Questionnaire within the Sleep Revolution consortium, encompassing non‐specific symptoms, and contextual and personal factors (Arnardottir et al. 2022).

This study has several limitations. First, we only included generic PRSMs, despite the availability of disorder‐specific PRSMs (e.g., for OSA, insomnia, RLS). Although this allowed for a more in‐depth analysis of generic PRSMs, future research could compare their content. Second, inter‐rater reliability was not assessed as coding was done by a single researcher (MDB). Third, while we detailed ICF categories per PRSM, presentation of item‐level linking was restricted due to licensing. Fourth, we used four semantic relationships to describe item concepts, though more may exist. Future studies could expand on our exploratory work with semantic analysis.

## 5 Conclusions

This study systematically mapped the content and relationships between concepts contained within PRSMs using the ICF and semantic analysis. PRSMs were found to insufficiently capture the biopsychosocial complexity of sleep disorders. Moreover, questionnaire labels did not always reflect their actual conceptual content. Future directions in sleep measurement should adopt a multidimensional perspective of sleep encompassing symptoms, the impact on daily functioning, and contextual factors.

## Author Contributions

**Marie De Bruecker:** conceptualization, investigation, writing – original draft, methodology, visualization, writing – review and editing, formal analysis, data curation. **Elke Veirman:** conceptualization, investigation, writing – original draft, methodology, validation, writing – review and editing, data curation, formal analysis, supervision. **An Mariman:** conceptualization, writing – review and editing, funding acquisition, supervision. **Dirk A. Pevernagie:** conceptualization, funding acquisition, writing – review and editing, methodology, project administration, supervision. **Katrien B. C. Hertegonne:** conceptualization, writing – review and editing, supervision, methodology. **Fré A. Bauters:** conceptualization, methodology, writing – review and editing, supervision. **Dimitri M. L. Van Ryckeghem:** conceptualization, methodology, writing – review and editing, supervision. **Geert Crombez:** conceptualization, writing – original draft, writing – review and editing, methodology, supervision, funding acquisition.

## Funding

This work was supported by Horizon 2020 Framework Programme (#965417) and Universiteit Gent (BOFBAF2024071501).

## Conflicts of Interest

The authors declare no conflicts of interest.

## Supporting information


**Table S1:** Excluded sleep disorder specific‐questionnaires.

**Table S2:** Validation populations of PRSMs.

**Table S3:** All mapped ICF categories of the 4th level.

**Table S4:** ICF content of all PRSMs.

## Data Availability

The data that support the findings of this study are available from the corresponding author upon reasonable request.
