# Supplementary material for: What Do Patient‐Reported Sleep Measures Assess? A Content Analysis
Source: J Sleep Res. 2026 Jun 8;35(5):e70382. doi: 10.1111/jsr.70382 (PMC13619044; doi:10.1111/jsr.70382)
Supplement: Supplementary file 1 [file JSR-35-e70382-s001.docx]

| **Table S4** |  |  |  |  |  |  |  |  |  |  |  |  |  |  |  |  |  |  |  |  |  |  |  |
| --- | --- | --- | --- | --- | --- | --- | --- | --- | --- | --- | --- | --- | --- | --- | --- | --- | --- | --- | --- | --- | --- | --- | --- |
| *ICF content of all PRSMs* |  |  |  |  |  |  |  |  |  |  |  |  |  |  |  |  |  |  |  |  |  |  |  |
| **Category** | **BFS** | **BQS** | **FACES** | **FSS** | **VAS-F** | **ISDI** | **LSEQ** | **MOS-SS** | **MaSQuDI-17** | **NRSS** | **PROMIS-SD SF** | **PROMIS-SRI SF** | **RSS** | **MOS-SS-R** | **SIQ** | **SQS** | **STQ** | **SWIFT** | **SWAI** | **ToDSS** | **THAT** | **VAS-T/S** | **ZOGIM-A** |
| Energy level | 8 | 4 | 34 | 8 | 22 | 13 | 4 | 4 |  | 3 |  | 4 | 1 | 4 | 1 | 5 |  | 12 | 8 |  | 1 | 1 |  |
| Onset of sleep |  | 18 |  |  |  | 21 | 7 | 3 | 2 |  | 1 |  | 11 | 3 |  | 3 | 1 |  | 8 | 8 |  |  |  |
| Quality of sleep |  |  |  |  |  | 6 | 4 | 1 |  | 4 | 3 | 3 |  | 1 |  | 12 |  |  | 1 |  |  |  |  |
| Maintenance of sleep |  | 2 |  |  |  | 20 | 2 | 2 | 1 |  | 1 |  |  | 2 | 1 | 8 |  |  | 2 |  |  |  |  |
| Functions involving the sleep cycle |  |  |  |  |  | 22 | 7 | 2 | 2 | 1 |  | 1 |  | 2 | 3 | 1 |  |  |  |  |  |  |  |
| Range of emotion |  |  |  |  |  | 2 |  |  | 6 | 3 |  | 1 |  |  | 2 | 1 |  |  | 5 |  |  |  |  |
| Continuity of consciousness |  | 4 | 1 |  |  | 2 | 2 |  |  | 1 |  | 1 |  |  |  |  |  |  |  |  | 2 | 1 | 10 |
| Amount of sleep |  |  |  |  |  | 4 |  | 3 |  | 1 | 1 |  |  | 3 | 1 | 2 | 1 |  |  |  |  |  | 1 |
| Sustaining attention | 2 | 4 |  |  | 2 | 2 |  |  | 1 | 1 |  | 1 |  |  | 1 |  |  |  |  |  | 2 |  |  |
| Sleep functions, unspecified |  | 2 |  |  |  |  |  | 1 |  |  | 2 |  |  | 1 |  | 3 |  |  |  |  |  |  |  |
| Sleep functions, other specified |  |  | 1 |  |  | 1 |  |  |  |  |  |  |  |  | 1 | 1 |  |  |  |  |  |  |  |
| Psychomotor control |  |  | 3 |  |  |  |  |  |  |  |  |  |  |  | 2 |  |  |  | 1 |  |  |  |  |
| State of consciousness |  |  | 5 |  |  |  |  |  |  |  |  |  |  |  | 1 |  |  |  |  |  |  |  |  |
| Emotional functions, other specified |  |  |  |  |  | 2 |  |  |  |  |  |  |  |  |  |  |  |  |  |  |  |  |  |
| Energy and drive functions, other specified |  | 8 |  |  | 4 |  |  |  |  |  |  |  |  |  |  | 1 |  |  |  |  |  |  |  |
| Motivation |  |  |  | 1 |  |  |  |  | 1 |  |  |  |  |  |  |  |  |  | 1 |  |  |  |  |
| Pace of thought |  |  |  |  |  | 1 |  |  | 1 |  |  |  |  |  | 1 |  |  |  | 2 |  |  |  |  |
| Control of thought |  |  |  |  |  |  |  |  | 2 |  |  |  |  |  |  |  |  |  |  |  |  |  |  |
| Content of thought |  |  |  |  |  | 2 |  |  | 1 |  |  |  |  |  |  |  |  |  |  |  |  |  |  |
| Retrieval and processing of memory |  |  |  |  |  |  |  |  |  |  |  |  |  |  |  | 2 |  |  |  |  |  |  |  |
| Regulation of emotion |  |  |  |  |  |  |  |  | 1 |  |  |  |  |  |  |  |  |  |  |  |  |  |  |
| Appetite |  |  |  |  |  |  |  |  |  |  |  |  |  |  |  | 1 |  |  | 1 |  |  |  |  |
| Form of thought |  |  |  |  |  |  |  |  |  |  |  |  |  |  | 1 | 1 |  |  |  |  |  |  |  |
| Agreeableness |  |  |  |  | 2 |  |  |  |  |  |  |  |  |  |  |  |  |  |  |  |  |  |  |
| Thought functions, other specified |  |  |  |  |  |  |  |  |  |  |  |  |  |  |  |  |  |  | 1 |  | 1 |  |  |
| Memory functions, unspecified |  |  |  |  |  |  |  |  |  | 1 |  |  |  |  |  |  |  |  |  |  |  |  |  |
| Emotional functions, unspecified |  |  |  |  |  | 1 |  |  |  |  |  |  |  |  |  |  |  |  |  |  |  |  |  |
| Orientation functions, unspecified |  |  |  |  |  |  |  |  |  |  |  |  |  |  |  |  |  |  |  |  |  |  |  |
| Extraversion |  |  |  |  |  |  |  |  |  |  |  |  |  |  |  |  |  |  | 1 |  |  |  |  |
| Mental functions | 1 |  |  |  |  |  |  |  |  |  |  |  |  |  |  |  |  |  |  |  |  |  |  |
| Temperament and personality functions, other specified |  |  | 1 |  |  |  |  |  |  |  |  |  |  |  |  |  |  |  |  |  |  |  |  |
| Psychic stability |  |  | 1 |  |  |  |  |  |  |  |  |  |  |  |  |  |  |  |  |  |  |  |  |
| Optimism |  |  | 1 |  |  |  |  |  |  |  |  |  |  |  |  |  |  |  |  |  |  |  |  |
| Consciousness functions |  |  |  |  |  |  |  |  |  |  |  |  |  |  | 1 |  |  |  |  |  |  |  |  |
| Pain in head and neck |  |  |  |  |  |  |  | 1 | 1 | 1 |  |  |  | 1 |  | 1 |  |  |  |  |  |  |  |
| *Pain in stomach or abdomen* |  |  |  |  |  |  |  |  | *1 (A)* |  |  |  |  |  |  |  |  |  |  |  |  |  |  |
| Sensitivity to temperature |  |  |  |  |  |  |  |  |  |  |  |  |  |  |  |  |  |  |  |  |  |  |  |
| Vestibular function of balance |  |  |  |  |  |  | 1 |  |  |  |  |  |  |  | 1 |  |  |  |  |  |  |  |  |
| Sensations associated with the eye and adjoining structures |  |  |  |  |  |  |  |  |  |  |  |  |  |  | 1 |  |  |  |  |  |  |  |  |
| Pain in lower limb |  |  |  |  |  | 1 |  |  |  |  |  |  |  |  |  |  |  |  |  |  |  |  |  |
| Seeing functions |  |  |  |  |  |  |  |  |  |  |  |  |  |  |  |  |  |  |  |  | 1 |  |  |
| Sensation of pain |  |  | 1 |  |  |  |  |  |  |  |  |  |  |  |  |  |  |  |  |  |  |  |  |
| Additional sensory functions, other specified and unspecified |  |  |  |  |  |  |  |  |  | 1 |  |  |  |  |  |  |  |  |  |  |  |  |  |
| Generalized pain |  |  |  |  |  |  |  |  |  | 1 |  |  |  |  |  |  |  |  |  |  |  |  |  |
| Touch function |  |  |  |  |  |  |  |  |  | 1 |  |  |  |  |  |  |  |  |  |  |  |  |  |
| Sensation of pain, other specified and unspecified |  |  |  |  |  |  |  |  |  |  |  |  |  |  |  |  |  |  |  |  |  |  |  |
| Sensations associated with cardiovascular and respiratory functions |  |  |  |  |  | *2 (A)* |  | 1 | *3 (2A)* | 3 |  |  |  | 1 | 1 |  |  |  | 1 |  |  |  |  |
| Additional functions of the respiratory system |  | 1 | 1 |  |  |  |  | 1 |  |  |  |  |  | 1 |  |  |  |  |  |  |  |  |  |
| Fatiguability |  |  |  | 1 |  |  |  |  | 1 |  |  |  |  |  |  |  |  |  |  |  |  |  |  |
| Functions of coughing |  |  |  |  |  |  |  |  |  | 1 |  |  |  |  |  |  |  |  |  |  |  |  |  |
| Respiratory rhythm |  |  |  |  |  |  |  |  |  |  |  |  |  |  |  |  |  |  |  |  |  |  |  |
| Respiration functions, unspecified |  |  |  |  |  |  |  |  |  |  |  |  |  |  |  |  |  |  |  |  |  |  |  |
| Sensation of nausea |  |  |  |  |  |  |  |  |  | 1 |  |  |  |  |  |  |  |  |  |  |  |  |  |
| Frequency of urination |  |  |  |  |  |  |  |  |  |  |  |  |  |  |  |  | 1 |  |  |  |  |  |  |
| Involuntary contractions of muscles |  |  |  |  |  | 8 |  | *1 (A)* |  |  |  |  |  |  |  |  |  |  |  |  |  |  |  |
| Movement functions, other specified and unspecified |  |  |  |  | 2 |  |  |  | 1 |  |  |  |  |  |  | 1 |  |  |  |  |  |  |  |
| Sensations related to muscles and movement functions, other specified |  |  |  |  |  | 3 |  |  | 1 |  |  |  |  |  |  |  |  |  |  |  |  |  |  |
| Muscle tone functions, unspecified |  |  |  |  |  |  |  | *1 (A)* |  |  |  |  |  |  | 1 |  |  |  |  |  |  |  |  |
| Control of voluntary movement functions, unspecified |  |  |  |  |  |  | 2 |  |  |  |  |  |  |  | 1 |  |  |  |  |  |  |  |  |
| Sensations related to muscles and movement functions |  |  |  |  |  |  |  |  |  |  |  |  |  |  | 2 |  |  |  |  |  |  |  |  |
| Sensations related to muscles and movement functions, unspecified |  |  |  |  |  | *1 (A)* |  |  |  |  |  |  |  |  |  |  |  |  |  |  |  |  |  |
| Sensation of muscle stiffness |  |  |  |  |  |  |  |  |  |  |  |  |  |  |  |  |  |  | 1 |  |  |  |  |
| Coordination of voluntary movements |  |  |  |  |  |  | 1 |  |  |  |  |  |  |  |  |  |  |  |  |  |  |  |  |
| Sensation of muscle spasm |  |  |  |  |  | 1 |  |  |  |  |  |  |  |  |  |  |  |  |  |  |  |  |  |
| Control of voluntary movement function |  |  |  |  |  |  |  |  |  |  |  |  |  |  |  |  |  |  |  |  |  |  |  |
| *Tremor* |  |  |  |  |  |  |  |  | *1 (A)* |  |  |  |  |  |  |  |  |  |  |  |  |  |  |
| Sensation related to the skin |  |  |  |  |  |  |  |  |  | 1 |  |  |  |  |  |  |  |  |  |  |  |  |  |
| *Other functions of the skin* |  |  |  |  |  | *1 (A)* |  |  | *1 (A)* |  |  |  |  |  |  |  |  |  |  |  |  |  |  |
| Reading | *1 (A)* | *4 (A)* |  |  |  |  |  |  |  |  |  |  | 1 |  |  |  |  | 2 | 1 | 1 |  |  |  |
| Watching | *1 (A)* | *4 (A)* |  |  |  | 1 |  |  |  |  |  |  | 1 |  |  |  |  |  | 1 | 1 |  |  |  |
| Thinking |  |  |  |  |  |  |  |  |  |  |  |  |  |  |  | 1 |  |  |  |  | 1 |  |  |
| Listening |  |  |  |  |  |  |  |  |  |  |  |  | 1 |  |  |  |  |  |  |  |  |  |  |
| *Calculating, unspecified* | *1 (A)* |  |  |  |  |  |  |  |  |  |  |  |  |  |  |  |  |  |  |  |  |  |  |
| *Writing* | *1 (A)* |  |  |  |  |  |  |  |  |  |  |  |  |  |  |  |  |  |  |  |  |  |  |
| Carrying out daily routine, unspecified |  |  |  |  |  | 1 |  |  |  |  |  | 1 |  |  | 1 |  |  |  | 2 |  |  |  |  |
| Carrying out multiple tasks |  |  |  |  |  |  |  |  |  |  |  |  |  |  |  |  |  | 3 |  |  |  |  |  |
| Managing daily routine |  |  |  |  |  |  |  |  |  |  |  |  |  |  |  |  |  |  | 1 |  |  |  | 1 |
| Undertaking single tasks, unspecified |  |  |  |  |  |  |  |  | 1 |  |  |  |  |  |  |  |  |  |  |  | 1 |  |  |
| Managing one's own activity level |  |  |  |  |  |  |  |  |  |  |  |  |  |  |  |  |  |  |  |  | 1 |  |  |
| Handling responsibilities |  |  |  | 1 |  |  |  |  |  |  |  |  |  |  |  |  |  |  |  |  |  |  |  |
| Carrying out daily routine, other specified |  |  |  |  |  |  |  |  |  |  |  |  |  |  |  |  |  |  | 1 |  |  |  |  |
| Completing multiple tasks |  |  |  |  |  |  |  |  |  |  |  |  |  |  |  |  |  |  |  |  |  |  | 1 |
| Conversing with one person | 1 |  |  |  |  |  |  |  |  |  |  |  |  |  |  |  |  |  |  | 1 |  |  |  |
| *Conversing with many people* |  | *4 (A)* |  |  |  |  |  |  |  |  |  |  |  |  |  |  |  |  |  |  |  |  |  |
| Sustaining a conversation |  |  |  |  | 2 |  |  |  |  |  |  |  |  |  |  |  |  |  |  |  |  |  |  |
| Conversation, unspecified |  |  |  |  |  |  |  |  |  |  |  |  |  |  |  |  |  |  | 1 |  |  |  |  |
| *Speaking, unspecified* |  |  |  |  |  |  |  | *1 (A)* |  |  |  |  |  |  |  |  |  |  |  |  |  |  |  |
| Maintaining a sitting position |  |  |  |  |  | 1 |  |  |  |  |  |  | 7 |  |  |  |  |  | 2 | 4 |  |  |  |
| Lying down |  |  |  |  | 2 |  |  |  |  |  |  |  | 1 |  |  |  |  |  |  | 1 |  |  |  |
| Driving motorized vehicles |  |  |  |  |  |  |  |  |  |  |  |  | 1 |  |  |  |  | 1 |  | 1 |  |  |  |
| Driving, unspecified |  |  |  |  |  |  |  |  |  |  |  |  |  |  |  |  |  | 2 | 1 |  | *1 (A)* |  |  |
| Using private motorized transportation |  |  |  |  |  |  |  |  |  |  |  |  | 1 |  |  |  |  |  | 1 | 1 |  |  |  |
| *Driving human-powered transportation* |  | *4 (A)* |  |  |  |  |  |  |  |  |  |  |  |  |  |  |  |  |  |  |  |  |  |
| Using public motorized transportation |  | *4 (A)* |  |  |  |  |  |  |  |  |  |  | 1 |  |  |  |  |  |  |  |  |  |  |
| Shifting the body's centre of gravity |  |  |  |  |  | 1 |  |  |  |  |  |  |  |  |  |  |  |  |  |  |  |  |  |
| Maintaining a lying position |  |  |  |  |  | 1 |  |  |  |  |  |  |  |  |  |  |  |  |  |  |  |  |  |
| Maintaining a body position, unspecified |  |  |  |  |  |  |  |  | 1 |  |  |  |  |  |  |  |  |  |  |  |  |  |  |
| Standing |  |  |  |  |  |  | 1 |  |  |  |  |  |  |  |  |  |  |  |  |  |  |  |  |
| Climbing | 1 |  |  |  |  |  |  |  |  |  |  |  |  |  |  |  |  |  |  |  |  |  |  |
| Carrying, moving and handling objects, other specified and unspecified |  |  |  |  |  |  |  |  |  |  |  |  |  |  | 1 |  |  |  |  |  |  |  |  |
| Eating |  | 4 *(+4A)* |  |  |  |  |  |  |  |  |  |  | 2 |  |  |  |  |  |  | 1 |  |  |  |
| Ensuring one’s physical comfort |  |  |  |  |  | 1 |  |  |  |  |  |  |  |  |  |  |  |  |  |  |  |  |  |
| Caring for hair | 1 |  |  |  |  |  |  |  |  |  |  |  |  |  |  |  |  |  |  |  |  |  |  |
| Assisting others, unspecified |  |  |  |  |  |  |  |  |  |  |  |  |  |  |  |  |  |  | 1 |  |  |  |  |
| Doing housework | 1 |  |  |  |  |  |  |  |  |  |  |  |  |  |  |  |  |  |  |  |  |  |  |
| *Cleaning cooking area and utensils* | 1 (A) |  |  |  |  |  |  |  |  |  |  |  |  |  |  |  |  |  |  |  |  |  |  |
| *Cleaning living area* | 1 (A) |  |  |  |  |  |  |  |  |  |  |  |  |  |  |  |  |  |  |  |  |  |  |
| *Using household appliances* | 1 (A) |  |  |  |  |  |  |  |  |  |  |  |  |  |  |  |  |  |  |  |  |  |  |
| *Shopping* | 1 (A) |  |  |  |  |  |  |  |  |  |  |  |  |  |  |  |  |  |  |  |  |  |  |
| *Taking care of plants, indoors and outdoors* | 1 (A) |  |  |  |  |  |  |  |  |  |  |  |  |  |  |  |  |  |  |  |  |  |  |
| Family relationships, unspecified |  |  |  | 1 |  |  |  |  | 1 |  |  |  |  |  |  |  |  |  |  |  |  |  |  |
| Interpersonal interactions and relationships, other specified |  |  |  |  |  |  |  |  |  |  |  |  |  |  |  | 1 |  |  |  |  |  |  |  |
| Remunerative employment, unspecified |  | *4 (A)* |  | 1 |  |  |  |  | 1 |  |  |  | 1 |  |  | 2 |  | 2 | 2 |  |  |  |  |
| Education unspecified |  |  |  |  |  |  |  |  |  |  |  |  | 1 |  |  |  |  | 2 |  |  |  |  |  |
| *School education* | 2 (A) |  |  |  |  |  |  |  |  |  |  |  |  |  |  |  |  |  |  |  |  |  |  |
| Remunerative employment, other specified |  |  |  |  |  |  |  |  |  |  |  |  |  |  |  | 2 |  |  |  |  |  |  |  |
| Socializing |  |  |  | 1 |  |  |  |  |  |  |  |  | 1 |  |  |  |  | 2 | 1 |  |  |  |  |
| Recreation and leisure, unspecified |  |  |  |  |  | 1 |  |  |  |  |  |  |  |  |  |  |  |  | 4 |  |  |  |  |
| Sports | *1 (A)* |  |  | 1 |  |  |  |  |  |  |  |  |  |  |  |  |  |  |  |  |  |  | 1 |
| Hobbies |  |  |  |  |  |  |  |  |  |  |  |  |  |  |  |  |  |  | 2 |  |  |  |  |
| Arts and culture |  |  |  |  |  |  |  |  |  |  |  |  | 2 |  |  |  |  |  |  | *1 (A)* |  |  |  |
| Recreation and leisure, other specified |  |  |  |  |  |  |  |  | 1 |  |  |  |  |  |  |  |  |  |  |  |  |  |  |
| Recreation and leisure |  |  |  |  |  |  |  |  |  |  |  |  |  |  |  |  |  |  |  |  |  |  | 1 |
| Drugs |  | 1 |  |  |  |  | 7 |  |  |  |  |  |  |  |  |  |  |  |  |  |  |  |  |
| General products and technology for communication | *1 (A)* | *4 (A)* |  |  |  | 1 |  |  |  |  |  |  | 1 |  |  |  |  |  | 1 | 1 |  |  |  |
| Assistive products and technology for personal use in daily living |  |  |  |  |  |  |  |  |  |  |  |  |  |  | 2 |  |  |  |  |  |  |  |  |
| Financial assets |  |  |  |  |  |  |  |  | 1 |  |  |  |  |  |  |  |  |  |  |  |  |  |  |
| Food |  |  |  |  |  |  |  |  |  |  |  |  |  |  |  |  |  |  |  |  |  |  | 1 |
| Sound, unspecified |  |  |  |  |  | 1 |  |  |  |  |  |  |  |  |  | 1 |  |  |  |  |  |  |  |
| Sound intensity |  |  |  |  |  | 1 |  |  |  |  |  |  |  |  |  |  |  |  |  |  |  |  |  |
| Immediate family |  |  |  |  |  |  |  |  |  |  |  |  |  |  |  |  |  |  |  |  |  |  |  |
| Attitudes, unspecified |  |  |  |  |  | 1 |  |  |  |  |  |  |  |  |  |  |  |  | 3 |  |  |  |  |
| Legal services |  |  |  |  |  |  |  |  |  |  |  |  |  |  |  |  |  |  | 1 |  |  |  |  |
| Personal factors |  | 1 |  |  |  | 1 |  |  |  |  | 1 |  |  |  |  | 1 |  |  | 14 |  |  |  |  |
| Concept not covered by ICF |  | 4 |  |  | 2 | 1 |  |  | 1 |  |  |  | 1 |  | 3 | 1 |  |  | 8 |  | 2 |  | 2 |
| Health condition |  |  |  |  |  |  |  |  |  |  |  |  |  |  |  |  |  |  |  |  |  |  |  |
| General health |  |  |  |  |  |  |  |  |  |  |  |  |  |  |  |  |  |  |  |  |  |  |  |
| Concept not defined by ICF | 1 | 8 | 1 | 1 | 2 | 3 |  |  | 1 |  |  |  |  |  |  |  | 16 |  | 4 |  | 1 |  | 1 |
| nd-func |  |  |  | 2 |  |  |  |  |  |  |  | 1 |  |  |  | 1 |  |  |  |  |  |  |  |
| nd-dis |  |  |  | 1 |  |  |  |  |  |  |  |  |  |  |  |  |  |  |  |  |  |  |  |
| nd-ph |  |  |  |  |  |  |  |  | 1 | 1 |  |  |  |  |  |  |  |  |  |  |  |  |  |
| nd-gh |  |  |  |  |  |  |  |  |  | 1 |  |  |  |  |  |  |  |  | 1 |  |  |  |  |
| nc-hc |  |  |  |  |  |  |  |  | 1 |  |  |  |  |  |  |  |  |  |  |  |  |  |  |

*Note*. Nd-func: not definable–functioning; nd-dis: not definable–disability; nd-ph: not definable-physical health; nd-gh: not definable-general health; nc-hc: not covered-health condition; A = additional concept. Categories solely used for linking additional concepts are presented in italics*.*
