# Supplementary material for: What Do Patient‐Reported Sleep Measures Assess? A Content Analysis
Source: J Sleep Res. 2026 Jun 8;35(5):e70382. doi: 10.1111/jsr.70382 (PMC13619044; doi:10.1111/jsr.70382)
Supplement: Supplementary file 2 [file JSR-35-e70382-s002.docx]

| **Table S2** | | | |
| --- | --- | --- | --- |
| *Validation populations of PRSMs* | | | |
| **#** | **Questionnaire** | **Acronym** | **Validated populations** |
| 1 | Brugmann Fatigue Scale [1] | BFS | **SRBD, SRMD, central disorders of hypersomnolence, fibromyalgia, and chronic fatigue syndrome (CFS).** |
| 2 | Bologna Questionnaire on Sleepiness-related Questions [2] | BQS | **Patients being evaluated for excessive daytime sleepiness (EDS), SRBD and other than sleep disorders** |
| 3 | Epworth Sleepiness Scale [3] | ESS | **SRBD, narcolepsy, idiopathic hypersomnia, insomnia, periodic limb movement disorder (PLMD)**, restless legs syndrome (RLS), healthy controls, insomnia, OSA [4], OSA, PLMD, rapid eye movement sleep behavior disorder (RBD) and narcolepsy [5], an obstetric population [6], stroke [7] |
| 4 | Fatigue, Anergy, Consciousness, Energized and Sleepiness [8] | FACES | **Insomnia** |
| 5 | Fatigue Severity Scale [9] | FSS | **Systemic lupus erythematosus and multiple sclerosis, healthy controls**, stroke, narcolepsy with cataplexy, RLS, OSA, insomnia, parasomnia, hypersomnia, other sleep-wake disorders [10], Parkinson’s disease [11] |
| 6 | Fatigue subscale of the Visual Analogue Scale [12] | VAS-F | **Patients with fatigue, healthy controls** |
| 7 | Iowa Sleep Disturbances Inventory [13] | ISDI | **Students, psychiatric patients (depression, anxiety disorders, substance use disorders, obsessive compulsive disorder), sleep-disorder patients (OSA, hypersomnia, insomnia, RLS, narcolepsy, PLMD, circadian rhythm, sleep disorder related to a mental condition).** |
| 8 | Leeds Sleep Evaluation Questionnaire [14] | LSEQ | **Volunteers using an active or placebo hypnotic drug,** insomnia [15], university students [16] |
| 9 | Medical Outcomes Study-Sleep Scale [17,18] | MOS-SS | **Chronic conditions, healthy controls,** **patients with neuropathic pain**, RLS [19], postmenopausal women [20], persons with overactive bladder [20], fibromyalgia [21] |
| 10 | Maugeri Sleep Quality and Distress Inventory [22] | MaSQuDI-17 | **Patients with OSA, insomnia, REM behavior disorders, parasomnia, PLMD, RLS, sleep-related eating disorders, central hypersomnia, healthy controls** |
| 11 | Nonrestorative sleep scale [23] | NRSS | **SRBD, insomnia, sleep disturbance due to psychiatric illness, PLMD, CRSWD, nonspecific sleep disturbance, parasomnia, narcolepsy, fibromyalgia, healthy controls** |
| 12 | Patient Reported Outcomes Measurement Information System (PROMIS) Item Bank v. 1.0 – Sleep Disturbance | PROMIS-SD [24] | **Self-reported sleep disorders, healthy controls, patients recruited from sleep medicine, general medicine, and psychiatric clinics**, Parkinson’s disease [25], traumatic brain injury (TBI) [26] |
| 13 | Patient Reported Outcomes Measurement Information System (PROMIS) Item Bank v. 1.0 – Sleep-related Impairments | PROMIS-SRI [24] | **Self-reported sleep disorders, healthy controls, patients recruited from sleep and general medicine, and psychiatric clinics,** Parkinson’s disease [25] |
| 14 | Pittsburgh Sleep Quality Index [27] | PSQI | **Healthy controls, major depressive disorder (MDD), disorders of initiating and maintaining sleep, disorders of EDS,** insomnia, RLS, OSA, healthy controls [4], insomnia [28], OSA [29], general population [30], OSA, PLMD, RBD and narcolepsy [5], university students [31], depression [32], OSA, depression and healthy controls [33], cancer patients [34–36], GAD [37], insomnia, EDS, narcolepsy, sleep bruxism [38], insomnia, narcolepsy [39], fibromyalgia [40], GAD, MDD, schizophrenia, insomnia, and controls [41], schizophrenia, anxiety and depressive disorder [42], women with hot flashes [43], temporomandibular disorders, pregnant women [44], systemic lupus erythematosus patients [45], patients with coronary heart disease, renal transplant recipients [46], shift workers [47] |
| 15 | PROMIS Sleep Disturbance Short Form [48] | PROMIS-SD SF | **Healthy controls, insomnia, OSA, RLS,** TBI [26], older adults from retirement communities [49], working civilian and military populations [50], fibromyalgia, myalgic encephalomyelitis/CFS [51], persons with psychosis [52] |
| 16 | PROMIS Sleep-related Impairment Short Form 8a [48] | PROMIS-SRI SF | **Healthy controls, insomnia, OSA, RLS**, encephalomyelitis/CFS [51], persons with psychosis [52] |
| 17 | Resistance to Sleepiness Scale [53] | RSS | **Insomnia and parasomnia, OSA, narcolepsy and hypersomnia** [53] |
| 18 | revised version of the Medical Outcomes Study Sleep Scale [54] | MOS Sleep-R | **General population, OSA, rheumatoid arthritis, osteoarthritis** |
| 19 | Sleep Inertia Questionnaire [55] | SIQ | **MDD, healthy controls,** idiopathic hypersomnia, narcolepsy [56] |
| 20 | Sleep Quality Scale [57] | SQS | **Healthy controls, university students, insomnia,** OSA [58] |
| 21 | Sleep Timing Questionnaire [59] | STQ | **Healthy controls, depression, insomnia, other sleep disorders, caregivers of Alzheimer or organ transplant patients** |
| 22 | Sleepiness-wakefulness Inability and Fatigue Test [60] | SWIFT | **Healthy controls, OSA, narcolepsy** |
| 23 | Sleep-Wake Activity Inventory [61] | SWAI | **Individuals being evaluated for EDS or snoring, college students** |
| 24 | Time of Day Sleepiness Scale [62] | ToDSS | **Patients with a variety of sleep complaints: snoring, breathing stops, sleepiness, and insomnia, OSA patients** |
| 25 | Toronto Hospital Alertness Test [63] | THAT | **OSA, EDS, narcolepsy, PLM, parasomnias, insomnia, sleep**  **problems associated with depression, fibromyalgia or CFS, and CRSWD, no sleep disorder,** healthy adults [64] |
| 26 | Visual Analog Scale for Tiredness/Sleepiness [65] | VAS-T/S | **Narcolepsy, OSA, insomnia, healthy controls** |
| 27 | ZOGIM-A Alertness Scale [63] | ZOGIM-A | **OSA, EDS, narcolepsy, PLMs, parasomnias, insomnia, sleep**  **problems associated with depression, fibromyalgia or CFS, and CRSWD, no sleep disorder** |

*Note*. Populations included in the development papers are indicated in bolt.

**References**

[1]	Mairesse O, Damen V, Newell J, Kornreich C, Verbanck P, Neu D. The Brugmann fatigue scale: an analogue to the epworth sleepiness scale to measure behavioral rest propensity. Behavioral Sleep Medicine 2019.

[2]	Rinaldi R, Vignatelli L, D’Alessandro R, Bassein L, Sforza E, Plazzi G, et al. Validation of Symptoms Related to Excessive Daytime Sleepiness. Neuroepidemiology 2001;20:248–56. https://doi.org/10.1159/000054798.

[3]	Johns MW. A new method for measuring daytime sleepiness: the Epworth sleepiness scale. Sleep 1991;14:540–5.

[4]	Beaudreau SA, Spira AP, Stewart A, Kezirian EJ, Lui L-Y, Ensrud K, et al. Validation of the Pittsburgh Sleep Quality Index and the Epworth Sleepiness Scale in older black and white women. Sleep Med 2012;13:36–42. https://doi.org/10.1016/j.sleep.2011.04.005.

[5]	Nishiyama T, Mizuno T, Kojima M, Suzuki S, Kitajima T, Ando KB, et al. Criterion validity of the Pittsburgh Sleep Quality Index and Epworth Sleepiness Scale for the diagnosis of sleep disorders. Sleep Med 2014;15:422–9. https://doi.org/10.1016/j.sleep.2013.12.015.

[6]	Baumgartel KL, Terhorst L, Conley YP, Roberts JM. Psychometric evaluation of the Epworth Sleepiness Scale in an obstetric population. Sleep Med 2013;14:116–21. https://doi.org/10.1016/j.sleep.2012.10.007.

[7]	Mills RJ, Koufali M, Sharma A, Tennant A, Young CA. Is the Epworth Sleepiness Scale Suitable for Use in Stroke? Top Stroke Rehabil 2013;20:493–9. https://doi.org/10.1310/tsr2006-493.

[8]	Shapiro CM, Flanigan M, Fleming JAE, Morehouse R, Moscovitch A, Plamondon J, et al. Development of an adjective checklist to measure five FACES of fatigue and sleepiness. J Psychosom Res 2002;52:467–73. https://doi.org/10.1016/S0022-3999(02)00407-5.

[9]	Krupp LB. The Fatigue Severity Scale. Arch Neurol 1989;46:1121. https://doi.org/10.1001/archneur.1989.00520460115022.

[10]	Valko PO, Bassetti CL, Bloch KE, Held U, Baumann CR. Validation of the Fatigue Severity Scale in a Swiss Cohort. Sleep 2008;31:1601–7. https://doi.org/10.1093/sleep/31.11.1601.

[11]	Siciliano M, Chiorri C, De Micco R, Russo A, Tedeschi G, Trojano L, et al. Fatigue in Parkinson’s disease: Italian validation of the Parkinson Fatigue Scale and the Fatigue Severity Scale using a Rasch analysis approach. Parkinsonism Relat Disord 2019;65:105–10. https://doi.org/10.1016/j.parkreldis.2019.05.028.

[12]	Lee KA, Hicks G, Nino-Murcia G. Validity and Reliability of a Scale to Assess Fatigue. vol. 36. 1991.

[13]	Koffel E, Watson D. Development and Initial Validation of the Iowa Sleep Disturbances Inventory. Assessment 2010;17:423–39. https://doi.org/10.1177/1073191110362864.

[14]	Parrott AC, Hindmarch I. Factor analysis of a sleep evaluation questionnaire. Psychol Med 1978;8:325–9. https://doi.org/10.1017/S0033291700014379.

[15]	Tarrasch R, Laudon M, Zisapel N. Cross‐cultural validation of the Leeds sleep evaluation questionnaire (LSEQ) in insomnia patients. Human Psychopharmacology: Clinical and Experimental 2003;18:603–10. https://doi.org/10.1002/hup.534.

[16]	Manzar MdD, Salahuddin M, Maru TT, Alghadir A, Anwer S, Bahammam AS, et al. Validation of the adapted Leeds sleep evaluation questionnaire in Ethiopian university students. Health Qual Life Outcomes 2018;16:49. https://doi.org/10.1186/s12955-018-0876-0.

[17]	Stewart AL, Ware JE. Measuring functioning and well-being : the medical outcomes study approach. Duke University Press; 1992.

[18]	Hays RD, Martin SA, Sesti AM, Spritzer KL. Psychometric properties of the Medical Outcomes Study Sleep measure. Sleep Med 2005;6:41–4. https://doi.org/10.1016/j.sleep.2004.07.006.

[19]	Allen RP, Kosinski M, Hill-Zabala CE, Calloway MO. Psychometric evaluation and tests of validity of the Medical Outcomes Study 12-item Sleep Scale (MOS sleep). Sleep Med 2009;10:531–9. https://doi.org/10.1016/j.sleep.2008.06.003.

[20]	Zagalaz-Anula N, Hita-Contreras F, Martínez-Amat A, Cruz-Díaz D, Lomas-Vega R. Psychometric properties of the medical outcomes study sleep scale in Spanish postmenopausal women. Menopause 2017;24:824–31. https://doi.org/10.1097/GME.0000000000000835.

[21]	Cappelleri JC, Bushmakin AG, McDermott AM, Dukes E, Sadosky A, Petrie CD, et al. Measurement properties of the Medical Outcomes Study Sleep Scale in patients with fibromyalgia. Sleep Med 2009;10:766–70. https://doi.org/10.1016/j.sleep.2008.09.004.

[22]	Morrone E, Sguazzin C, Bertolotti G, Giordano A, Braghiroli A, Balestroni GL, et al. Development and validation of the Maugeri Sleep Quality and Distress Inventory (MaSQuDI-17). PLoS One 2017;12:e0180743. https://doi.org/10.1371/journal.pone.0180743.

[23]	Wilkinson K, Shapiro C. Development and Validation of the Nonrestorative Sleep Scale (NRSS). Journal of Clinical Sleep Medicine 2013;09:929–37. https://doi.org/10.5664/jcsm.2996.

[24]	Buysse DJ, Yu L, Moul DE, Germain A, Stover A, Dodds NE, et al. Development and validation of patient-reported outcome measures for sleep disturbance and sleep-related impairments. Sleep 2010;33:781–92.

[25]	Jones J, Nielson SA, Trout J, Swenson M, Reiley J, Tanner J, et al. A Validation Study of PROMIS Sleep Disturbance (PROMIS-SD) and Sleep Related Impairment (PROMIS-SRI) Item Banks in Individuals with Idiopathic Parkinson’s Disease and Matched Controls. J Parkinsons Dis 2021;11:877–83. https://doi.org/10.3233/JPD-202429.

[26]	Carlozzi NE, Ianni PA, Tulsky DS, Brickell TA, Lange RT, French LM, et al. Understanding Health-Related Quality of Life in Caregivers of Civilians and Service Members/Veterans With Traumatic Brain Injury: Establishing the Reliability and Validity of PROMIS Fatigue and Sleep Disturbance Item Banks. Arch Phys Med Rehabil 2019;100:S102–9. https://doi.org/10.1016/j.apmr.2018.05.020.

[27]	Buysse DJ, Reynolds CF, Monk TH, Berman SR, Kupfer DJ. The Pittsburgh sleep quality index: A new instrument for psychiatric practice and research. Psychiatry Res 1989;28:193–213. https://doi.org/10.1016/0165-1781(89)90047-4.

[28]	Dudysová D, Malá I, Mladá K, Saifutdinova E, Koprivova J, Sos P. Structural and construct validity of the Czech version of the Pittsburgh Sleep Quality Index in chronic insomnia. Neuro Endocrinol Lett 2017;38:67–73.

[29]	Shochat T, Tzischinsky O, Oksenberg A, Peled R. Validation of the Pittsburgh Sleep Quality Index Hebrew translation (PSQI-H) in a sleep clinic sample. Isr Med Assoc J 2007;9:853–6.

[30]	Hinz A, Glaesmer H, Brähler E, Löffler M, Engel C, Enzenbach C, et al. Sleep quality in the general population: psychometric properties of the Pittsburgh Sleep Quality Index, derived from a German community sample of 9284 people. Sleep Med 2017;30:57–63. https://doi.org/10.1016/j.sleep.2016.03.008.

[31]	Aloba OO, Adewuya AO, Ola BA, Mapayi BM. Validity of the Pittsburgh Sleep Quality Index (PSQI) among Nigerian university students. Sleep Med 2007;8:266–70. https://doi.org/10.1016/j.sleep.2006.08.003.

[32]	Anandakumar D, Dayabandara M, Ratnatunga SS, Hanwella R, De Silva VA. Validation of the Sinhala version of the Pittsburgh Sleep Quality Index. Ceylon Medical Journal 2016;61:22. https://doi.org/10.4038/cmj.v61i1.8255.

[33]	Popević MB, Milovanović APS, Milovanović S, Nagorni-Obradović L, Nešić D, Velaga M. Reliability and Validity of the Pittsburgh Sleep Quality Index-Serbian Translation. Eval Health Prof 2018;41:67–81. https://doi.org/10.1177/0163278716678906.

[34]	Beck SL, Schwartz AL, Towsley G, Dudley W, Barsevick A. Psychometric evaluation of the Pittsburgh sleep quality index in cancer patients. J Pain Symptom Manage 2004;27:140–8. https://doi.org/10.1016/j.jpainsymman.2003.12.002.

[35]	Fontes F, Gonçalves M, Maia S, Pereira S, Severo M, Lunet N. Reliability and validity of the Pittsburgh Sleep Quality Index in breast cancer patients. Supportive Care in Cancer 2017;25:3059–66. https://doi.org/10.1007/s00520-017-3713-9.

[36]	Kotronoulas GC, Papadopoulou CN, Papapetrou A, Patiraki E. Psychometric evaluation and feasibility of the Greek Pittsburgh Sleep Quality Index (GR-PSQI) in patients with cancer receiving chemotherapy. Supportive Care in Cancer 2011;19:1831–40. https://doi.org/10.1007/s00520-010-1025-4.

[37]	Bush AL, Armento MEA, Weiss BJ, Rhoades HM, Novy DM, Wilson NL, et al. The Pittsburgh Sleep Quality Index in older primary care patients with generalized anxiety disorder: Psychometrics and outcomes following cognitive behavioral therapy. Psychiatry Res 2012;199:24–30. https://doi.org/10.1016/j.psychres.2012.03.045.

[38]	Gomes AA, Marques DR, Meiavia AM, Cunha F, Clemente V. Psychometric properties and accuracy of the European Portuguese version of the Pittsburgh Sleep Quality Index in clinical and non-clinical samples. Sleep Biol Rhythms 2018;16:413–22. https://doi.org/10.1007/s41105-018-0171-9.

[39]	Sohn S Il, Kim DH, Lee MY, Cho YW. The reliability and validity of the Korean version of the Pittsburgh Sleep Quality Index. Sleep and Breathing 2012;16:803–12. https://doi.org/10.1007/s11325-011-0579-9.

[40]	Hita-Contreras F, Martínez-López E, Latorre-Román PA, Garrido F, Santos MA, Martínez-Amat A. Reliability and validity of the Spanish version of the Pittsburgh Sleep Quality Index (PSQI) in patients with fibromyalgia. Rheumatol Int 2014;34:929–36. https://doi.org/10.1007/s00296-014-2960-z.

[41]	Farrahi Moghaddam J, Nakhaee N, Sheibani V, Garrusi B, Amirkafi A. Reliability and validity of the Persian version of the Pittsburgh Sleep Quality Index (PSQI-P). Sleep and Breathing 2012;16:79–82. https://doi.org/10.1007/s11325-010-0478-5.

[42]	Takács J, Bódizs R, Ujma PP, Horváth K, Rajna P, Harmat L. Reliability and validity of the Hungarian version of the Pittsburgh Sleep Quality Index (PSQI-HUN): comparing psychiatric patients with control subjects. Sleep and Breathing 2016;20:1045–51. https://doi.org/10.1007/s11325-016-1347-7.

[43]	Otte JL, Rand KL, Landis CA, Paudel ML, Newton KM, Woods N, et al. Confirmatory factor analysis of the Pittsburgh Sleep Quality Index in women with hot flashes. Menopause 2015;22:1190–6. https://doi.org/10.1097/GME.0000000000000459.

[44]	Zhong Q-Y, Gelaye B, Sánchez SE, Williams MA. Psychometric Properties of the Pittsburgh Sleep Quality Index (PSQI) in a Cohort of Peruvian Pregnant Women. Journal of Clinical Sleep Medicine 2015;11:869–77. https://doi.org/10.5664/jcsm.4936.

[45]	Magro R, Camilleri L, Borg AA. Translation and validation of the Fatigue Severity Scale, Pittsburgh Sleep Quality Index and Modified Health Assessment Questionnaire into the Maltese Language, in a cohort of Maltese Systemic Lupus Erythematosus patients. Mediterr J Rheumatol 2017;28:192–200. https://doi.org/10.31138/mjr.28.4.192.

[46]	Burkhalter H, Sereika SM, Engberg S, Wirz-Justice A, Steiger J, De Geest S. Structure validity of the Pittsburgh Sleep Quality Index in renal transplant recipients: A confirmatory factor analysis. Sleep Biol Rhythms 2010;8:274–81. https://doi.org/10.1111/j.1479-8425.2010.00473.x.

[47]	Roche J, Vos AG, Lalla-Edward ST, Kamerman PR, Venter WDF, Scheuermaier K. Importance of testing the internal consistency and construct validity of the Pittsburgh Sleep Quality Index (PSQI) in study groups of day and night shift workers: Example of a sample of long-haul truck drivers in South Africa. Appl Ergon 2022;98:103557. https://doi.org/10.1016/j.apergo.2021.103557.

[48]	Yu L, Buysse DJ, Germain A, Moul DE, Stover A, Dodds NE, et al. Development of Short Forms From the PROMIS^TM^ Sleep Disturbance and Sleep-Related Impairment Item Banks. Behavioral Sleep Medicine 2012;10:6–24. https://doi.org/10.1080/15402002.2012.636266.

[49]	Full KM, Malhotra A, Crist K, Moran K, Kerr J. Assessing psychometric properties of the PROMIS Sleep Disturbance Scale in older adults in independent-living and continuing care retirement communities. Sleep Health 2019;5:18–22. https://doi.org/10.1016/j.sleh.2018.09.003.

[50]	Brossoit RM, Stark HP, Crain TL, Bodner TE, Hammer LB, Mohr CD, et al. Multidimensionality of the PROMIS sleep disturbance 8b short form in working adult populations. Sleep Health 2023;9:925–32. https://doi.org/10.1016/j.sleh.2023.08.001.

[51]	Yang M, Keller S, Lin J-MS. Assessing sleep and pain among adults with myalgic encephalomyelitis/chronic fatigue syndrome: psychometric evaluation of the PROMIS® sleep and pain short forms. Quality of Life Research 2022;31:3483–99. https://doi.org/10.1007/s11136-022-03199-8.

[52]	Savage CLG, Orth RD, Jacome AM, Bennett ME, Blanchard JJ. Assessing the psychometric properties of the PROMIS sleep measures in persons with psychosis. Sleep 2021;44. https://doi.org/10.1093/sleep/zsab140.

[53]	Violani C, Lucidi F, Robusto E, Devoto A, Zucconi M, Ferini Strambi L. The assessment of daytime sleep propensity: a comparison between the Epworth Sleepiness Scale and a newly developed Resistance to Sleepiness Scale. Clinical Neurophysiology 2003;114:1027–33. https://doi.org/10.1016/S1388-2457(03)00061-0.

[54]	Yarlas A, White MK, St. Pierre DG, Bjorner JB. The development and validation of a revised version of the Medical Outcomes Study Sleep Scale (MOS Sleep-R). J Patient Rep Outcomes 2021;5:40. https://doi.org/10.1186/s41687-021-00311-3.

[55]	Kanady JC, Harvey AG. Development and Validation of the Sleep Inertia Questionnaire (SIQ) and Assessment of Sleep Inertia in Analogue and Clinical Depression. Cognit Ther Res 2015;39:601–12. https://doi.org/10.1007/s10608-015-9686-4.

[56]	Sung E-R, Maness CB, Cook JD, Vascan AM, Moron D, Saini P, et al. Validation and performance of the sleep inertia questionnaire in central disorders of hypersomnolence. Sleep Med 2024;121:352–8. https://doi.org/10.1016/j.sleep.2024.07.024.

[57]	YI H, SHIN K, SHIN C. Development of the Sleep Quality Scale. J Sleep Res 2006;15:309–16. https://doi.org/10.1111/j.1365-2869.2006.00544.x.

[58]	Yi H, Shin K, Kim J, Kim J, Lee J, Shin C. Validity and reliability of Sleep Quality Scale in subjects with obstructive sleep apnea syndrome. J Psychosom Res 2009;66:85–8. https://doi.org/10.1016/j.jpsychores.2008.07.008.

[59]	Monk TH, Buysse DJ, Kennedy KS, Potts JM, DeGrazia JM, Miewald JM. Measuring Sleep Habits Without Using a Diary: The Sleep Timing Questionnaire. Sleep 2003;26:208–12. https://doi.org/10.1093/sleep/26.2.208.

[60]	Sangal RB. Evaluating Sleepiness-Related Daytime Function by Querying Wakefulness Inability and Fatigue: Sleepiness-Wakefulness Inability and Fatigue Test (SWIFT). Journal of Clinical Sleep Medicine 2012;08:701–11. https://doi.org/10.5664/jcsm.2270.

[61]	Rosenthal L, Roehrs TA, Roth T. The sleep-wake activity inventory: A self-report measure of daytime sleepiness. Biol Psychiatry 1993;34:810–20. https://doi.org/10.1016/0006-3223(93)90070-T.

[62]	Dolan DC, Taylor DJ, Okonkwo R, Becker PM, Jamieson AO, Schmidt-Nowara W, et al. The Time of Day Sleepiness Scale to assess differential levels of sleepiness across the day. J Psychosom Res 2009;67:127–33. https://doi.org/10.1016/j.jpsychores.2009.03.014.

[63]	Shapiro CM, Auch C, Reimer M, Kayumov L, Heslegrave R, Huterer N, et al. A new approach to the construct of alertness. J Psychosom Res 2006;60:595–603. https://doi.org/10.1016/j.jpsychores.2006.04.012.

[64]	Li S, Fong DYT, Wong JYH, Wilkinson K, Shapiro C, Choi EPH, et al. Psychometric evaluation of the Chinese version of the Toronto Hospital Alertness Test. J Patient Rep Outcomes 2020;4:32. https://doi.org/10.1186/s41687-020-00197-7.

[65]	Schneider C, Fulda S, Schulz H. Daytime variation in performance and tiredness/sleepiness ratings in patients with insomnia, narcolepsy, sleep apnea and normal controls. J Sleep Res 2004;13:373–83. https://doi.org/10.1111/j.1365-2869.2004.00427.x.
