# Supplementary material for: What Do Patient‐Reported Sleep Measures Assess? A Content Analysis
Source: J Sleep Res. 2026 Jun 8;35(5):e70382. doi: 10.1111/jsr.70382 (PMC13619044; doi:10.1111/jsr.70382)
Supplement: Supplementary file 3 [file JSR-35-e70382-s003.docx]

**Table S3**

*All mapped ICF categories of the 4th level*

| **Component** | **Chapter** | **Category** | **Number of times a concept**  **was linked to the category** |
| --- | --- | --- | --- |
| Body functions | b1 | Energy level | 147 |
|  |  | Onset of sleep | 108 |
|  |  | Quality of sleep | 58 |
|  |  | Maintenance of sleep | 52 |
|  |  | Functions involving the sleep cycle | 47 |
|  |  | Range of emotion | 23 |
|  |  | Continuity of consciousness | 25 |
|  |  | Amount of sleep | 19 |
|  |  | Sustaining attention | 18 |
|  |  | Sleep functions, unspecified | 13 |
|  |  | Energy and drive functions, other specified | 13 |
|  |  | Sleep functions, other specified | 7 |
|  |  | Psychomotor control | 6 |
|  |  | State of consciousness | 6 |
|  |  | Emotional functions, other specified | 5 |
|  |  | Pace of thought | 5 |
|  |  | Motivation | 4 |
|  |  | Control of thought | 3 |
|  |  | Content of thought | 3 |
|  |  | Retrieval and processing of memory | 2 |
|  |  | Regulation of emotion | 2 |
|  |  | Appetite | 2 |
|  |  | Form of thought | 2 |
|  |  | Agreeableness | 2 |
|  |  | Thought functions, other specified | 2 |
|  |  | Memory functions, unspecified | 1 |
|  |  | Emotional functions, unspecified | 1 |
|  |  | Orientation functions, unspecified | 1 |
|  |  | Extraversion | 1 |
|  |  | Mental functions | 1 |
|  |  | Temperament and personality functions, other specified | 1 |
|  |  | Psychic stability | 1 |
|  |  | Optimism | 1 |
|  |  | Consciousness functions | 1 |
|  | b2 | Pain in head and neck | 5 |
|  |  | *Pain in stomach or abdomen* | 1 |
|  |  | Sensitivity to temperature | 2 |
|  |  | Vestibular function of balance | 2 |
|  |  | Sensations associated with the eye and adjoining structures | 1 |
|  |  | Pain in lower limb | 1 |
|  |  | Seeing functions | 1 |
|  |  | Sensation of pain | 1 |
|  |  | Additional sensory functions, other specified and unspecified | 1 |
|  |  | Generalized pain | 1 |
|  |  | Touch function | 1 |
|  |  | Sensation of pain, other specified and unspecified | 1 |
|  | b4 | Sensations associated with cardiovascular and respiratory functions | 8 *(+ 4A)* |
|  |  | Additional functions of the respiratory system | 6 |
|  |  | Fatiguability | 2 |
|  |  | Functions of coughing | 2 |
|  |  | Respiratory rhythm | 1 |
|  |  | Respiration functions, unspecified | 1 |
|  | b5 | Sensation of nausea | 1 |
|  | b6 | Frequency of urination | 2 |
|  | b7 | Involuntary contractions of muscles | 10 *(+1A)* |
|  |  | *Tremor* | 1 |
|  |  | Movement functions, other specified and unspecified | 5 |
|  |  | Sensations related to muscles and movement functions, other specified | 4 |
|  |  | Muscle tone functions, unspecified | 2 *(+1A)* |
|  |  | Control of voluntary movement functions, unspecified | 2 |
|  |  | Sensations related to muscles and movement functions | 2 |
|  |  | Sensations related to muscles and movement functions, unspecified | 1 *(+1A)* |
|  |  | Sensation of muscle stiffness | 1 |
|  |  | Coordination of voluntary movements | 1 |
|  |  | Sensation of muscle spasm | 1 |
|  |  | Control of voluntary movement functions | 1 |
|  | b8 | Sensation related to the skin | 1 |
|  |  | Other functions of the skin | 2 *(+2A)* |
| Activities and participation | d1 | Reading | 6 *(+5A)* |
|  |  | Watching | 5 *(+5A)* |
|  |  | Thinking | 2 |
|  |  | Listening | 1 |
|  |  | *Calculating, unspecified* | 1 |
|  |  | *Writing* | 1 *(+1A)* |
|  | d2 | Carrying out daily routine, unspecified | 8 |
|  |  | Carrying out multiple tasks | 3 |
|  |  | Managing daily routine | 2 |
|  |  | Undertaking single tasks, unspecified | 2 |
|  |  | Managing one's own activity level | 1 |
|  |  | Handling responsibilities | 1 |
|  |  | Carrying out daily routine, other specified | 1 |
|  |  | Completing multiple tasks | 1 |
|  | d3 | *Speaking, unspecified* | 1 |
|  |  | Conversing with one person | 3 |
|  |  | *Conversing with many people* | *4* |
|  |  | Sustaining a conversation | 2 |
|  |  | Conversation, unspecified | 1 |
|  | d4 | Maintaining a sitting position | 18 |
|  |  | Lying down | 5 |
|  |  | Driving motorized vehicles | 4 |
|  |  | Driving, unspecified | 4 *(+1A)* |
|  |  | Using private motorized transportation | 4 |
|  |  | *Driving human-powered transportation* | *4* |
|  |  | Using public motorized transportation | 1 *(+4A)* |
|  |  | Shifting the body's centre of gravity | 1 |
|  |  | Maintaining a lying position | 1 |
|  |  | Maintaining a body position, unspecified | 1 |
|  |  | Standing | 1 |
|  |  | Climbing | 1 |
|  |  | Carrying, moving and handling objects, other specified and unspecified | 1 |
|  | d5 | Eating | 9 *(+4A)* |
|  |  | Ensuring one’s physical comfort | 2 |
|  |  | Caring for hair | 1 |
|  | d6 | *Cleaning cooking area and utensils* | *1* |
|  |  | *Cleaning living area* | *1* |
|  |  | *Using household appliances* | *1* |
|  |  | *Shopping* | *1* |
|  |  | *Taking care of plants, indoors and outdoors* | *1* |
|  |  | Assisting others, unspecified | 1 |
|  |  | Doing housework | 1 |
|  |  | Domestic life | 1 |
|  | d7 | Family relationships, unspecified | 2 |
|  |  | Interpersonal interactions and relationships, other specified | 1 |
|  | d8 | Remunerative employment, unspecified | 9 *(+4A)* |
|  |  | Education unspecified | 3 |
|  |  | Remunerative employment, other specified | 2 |
|  |  | *School education* | *2* |
|  | d9 | Socializing | 6 |
|  |  | Recreation and leisure, unspecified | 5 |
|  |  | Sports | 2 *(+1A)* |
|  |  | Hobbies | 2 |
|  |  | Arts and culture | 2 *(+1A)* |
|  |  | Recreation and leisure, other specified | 1 |
|  |  | Recreation and leisure | 1 |
| Environmental factors | e1 | Drugs | 9 |
|  |  | General products and technology for communication | 5 *(+5A)* |
|  |  | Assistive products and technology for personal use in daily living | 2 |
|  |  | Financial assets | 1 |
|  |  | Food | 1 |
|  | e2 | Sound, unspecified | 2 |
|  |  | Sound intensity | 1 |
|  | e3 | Immediate family | 6 |
|  | e4 | Attitudes, unspecified | 4 |
|  | e5 | Legal services | 1 |

*Note*. b1 = mental functions; b2 = sensory functions and pain; b4 = functions of the cardiovascular, haematological, immunological and respiratory systems; b5 = functions of the digestive, metabolic and endocrine systems; b6 = genitourinary and reproductive functions, b7 = neuromusculoskeletal and movement-related functions; b8 = functions of the skin and related structures; d1 = learning and applying knowledge; d2 = general tasks and demands; d3 = communication; d4 = mobility; d5 = self-care; d6 = domestic life; d7 = interpersonal interactions and relationships; d8 = major life areas; d9 = community, social and civic life; e1 = products and technology; e2 = Natural environment and human-made change to environment; e3 = support and relationships; e4 = attitudes; e5 = services, systems, policies; A = additional concepts. Categories solely used for linking additional concepts are presented in italics.
