# Supplementary material for: What Do Patient‐Reported Sleep Measures Assess? A Content Analysis
Source: J Sleep Res. 2026 Jun 8;35(5):e70382. doi: 10.1111/jsr.70382 (PMC13619044; doi:10.1111/jsr.70382)
Supplement: Supplementary file 4 [file JSR-35-e70382-s004.docx]

This table lists the excluded sleep disorder specific-questionnaires, more specifically insomnia-specific, restless legs-specific, sleep-related breathing disorder or obstructive sleep apnea-specific, hypersomnia or narcolepsy-specific and parasomnia-specific questionnaires. Sleep disorder specific-questionnaires are developed for the use in or have a primary focus on particular sleep disorders. If it is not clearly mentioned that a questionnaire is developed for a particular sleep disorder, the context of the development article makes this clear. For example, the introduction of the development article elaborates on insomnia disorder. Reasons of exclusion based on one of these criteria are described, based on information from the development paper of the questionnaires.

Table S1: Excluded sleep disorder specific-questionnaires

*Insomnia-specific questionnaires*

|  | **Questionnaire** | **Acronym** | **Reason of exclusion** |
| --- | --- | --- | --- |
| 1 | Anxiety and Preoccupation about Sleep Questionnaire [1] | APSQ | Developed for the use in insomnia: the APSQ was created to evaluate worry in insomnia disorder. |
| 2 | Athens Insomnia Scale [2] | AIS | Developed for the use in insomnia: the AIS was developed to measure insomnia disorder severity based on the criteria of the International Classification of Diseases, 10th Revision diagnostic criteria for insomnia. |
| 3 | Athens Insomnia Scale Brief Five-item Version [2] | AIS-5 | Developed for the use in insomnia: the AIS-5 is a short version of the AIS. |
| 4 | Daytime Insomnia Symptom Response Scale [3] | DISR | Developed for the use in insomnia: the DISR is an insomnia disorder-specific questionnaire designed to assess rumination. |
| 5 | Dysfunctional Beliefs and Attitudes about Sleep Scale [4,5] | DBAS | Developed for the use in insomnia: the DBAS was formulated to evaluate sleep-related beliefs and attitudes in people with insomnia disorder. |
| 6 | 16-item Dysfunctional Beliefs and Attitudes about Sleep Scale [6] | DBAS-16 | Developed for the use in insomnia: the DBAS-16 is a short version of the DBAS. |
| 7 | 10-item Dysfunctional Beliefs and Attitudes about Sleep Scale [7] | DBAS-10 | Developed for the use in insomnia: the DBAS-10 is a short version of the DBAS. |
| 8 | Dysfunctional Beliefs about Sleep-2 items [8] | DBS-2 | Developed for the use in insomnia: the DBS-2 was created to assess dysfunctional beliefs about sleep in individuals with insomnia disorder. |
| 9 | Flinders Fatigue Scale [9] | FFS | Developed for the use in insomnia: the FSS was developed to measure daytime fatigue in insomnia disorder. |
| 10 | Glasgow Content of Thoughts Inventory [10] | GCTI | Developed for the use in insomnia: the GCTI serves to evaluate cognitive processes occurring before sleep in people with insomnia disorder. |
| 11 | Glasgow Sleep Effort Scale [11] | GSES | Developed for the use in insomnia: the GSES was designed to gauge sleep effort - the tendency to control sleep - in people with insomnia disorder. |
| 12 | Glasgow Sleep Impact Index [12] | GSII | Developed for the use in insomnia: The GSII is a PROM developed to capture quality of life impairments related to sleep in insomnia disorder. |
| 13 | Hotel Dieu 16 [13] | HD-16 | Developed for the use in insomnia: the HD-16 is an insomnia-specific questionnaire constructed to evaluate quality of life. |
| 14 | Insomnia Catastrophizing Scale [14] | ICS | Developed for the use in insomnia: the ICS was created to target catastrophizing specially related to insomnia disorder. |
| 15 | Insomnia Daytime Symptoms and Impacts Questionnaire [15] | IDSIQ | Developed for the use in insomnia: The IDSIQ is a PROM intended to evaluate daytime symptoms associated with insomnia. |
| 16 | Insomnia Diurnal Impact Scale [16] | IDIS | Developed for the use in insomnia: the IDIS is a PROM constructed to measure the daytime impact of insomnia disorder. |
| 17 | Insomnia Severity Index [17] | ISI | Developed for the use in insomnia: the ISI was developed to quantify the severity and impact of insomnia complaints. The ISI is also used to detect cases of insomnia disorder by grading disorder severity. |
| 18 | 2-item ISI version [18] | ISI-2 | Developed for the use in insomnia: the ISI-2 is a selection of two items from the ISI. |
| 19 | Insomnia Worry Questionnaire [19] | IWQ | Developed for the use in insomnia: The IWQ was created to measure worrying during the night in individuals with insomnia disorder. |
| 20 | Lebanese Insomnia Scale [20] | LIS-18 | Developed for the use in insomnia: the LIS-18 was developed to evaluate a range of symptoms and aspects of insomnia disorder. |
| 21 | Metacognitions Questionnaire – Insomnia [21] | MCQ-I | Developed for the use in insomnia: the MCQ-I serves to assess metacognitive beliefs in people with insomnia disorder. |
| 22 | Nighttime Thoughts Questionnaire [22] | NTTQ | Developed for the use in insomnia: the NTTQ aims to capture nighttime thoughts and worries in insomnia disorder. |
| 23 | Occupational Impact of Sleep Questionnaire [23] | OISQ | Developed for the use in insomnia: the OISQ was developed to evaluate the occupational impact of insomnia disorder. |
| 24 | Pittsburgh Insomnia Rating Scale [24] | PIRS | Developed for the use in insomnia: the PIRS is an insomnia disorder-specific item bank aiming to provide a comprehensive assessment of symptoms. |
| 25 | 2-item Pittsburgh Insomnia Rating Scale [24] | PIRS2 | Developed for the use in insomnia: the PIRS2 is a selection of two items from the PIRS. |
| 26 | Pre-sleep Arousal Scale [25] | PSAS | Context: the PSAS was developed to assess pre-sleep cognitive and somatic arousal. Although not explicitly stated that the PSAS is an insomnia-specific scale, the introduction highlights these arousal processes as central to insomnia, and the validation focused on distinguishing people with insomnia disorder from good sleepers. The authors also emphasize the potential of the PSAS as an outcome measure in insomnia-specific treatment studies, and for assessing pre-sleep arousal across different insomnia subtypes. |
| 27 | Regensburg Insomnia Scale  [26] | RIS | Developed for the use in insomnia: the RIS was create to examine cognitive, emotional and behavioural aspects of insomnia disorder. |
| 28 | Restorative Sleep Questionnaire [27] | RSQ | Developed for the use in insomnia: the RSQ is intended to characterize nonrestorative sleep among individuals with insomnia disorder. |
| 29 | Self-statement test: 60+ [28] | SST:60+ | Context: the SST:60+ was designed to target pre-sleep related thoughts experienced by older individuals. Although the SST:60+ is not explicitly labelled as an insomnia-specific questionnaire, the development paper frames the SST:60+ within the context of insomnia disorder in older adults, and conceptualizes these cognitive processes as a core symptom. |
| 30 | Sleep Associated Monitoring Index [29] | SAMI | Context: the SAMI was created to index monitoring for sleep-related threat. Although not specifically stated as an insomnia-specific questionnaire, monitoring processes are described a perpetuating factors of insomnia, and the authors position the SAMI as an outcome measure for experimental and treatment-outcome studies in insomnia disorder. |
| 31 | Sleep Acceptance Scale [30] | SAS | Context: the SAS is a tool constructed to measure sleep acceptance by capturing cognitive and emotional responses to sleep problems. Although not explicitly stated as an insomnia-specific questionnaire, the development of the SAS is framed within the context of insomnia disorder, highlighting the relevance of acceptance and avoidance processes in the treatment and understanding of insomnia. |
| 32 | Sleep Behavior Self-Rating Scale [31] | SBSRS | Context: the SBSRS was developed to assess sleep-incompatible behaviours. Although the SBSRS is not specially mentioned as an insomnia-specific questionnaire, it is described how these behaviours are associated with an insomniac’s sleep environment. In the validation process, the SBSRS also differentiates individuals with and without insomnia disorder. |
| 33 | Sleep Disturbance Questionnaire [32] | SDQ | Developed for the use in insomnia: the SDQ was designed to identify attributions regarding the sources of sleep problems across physiological, behavioural and cognitive domains in individuals with insomnia disorder. |
| 34 | Sleep Dissatisfaction Questionnaire [33] | SDsQ | Developed for the use in insomnia: the SDsQ is an expanded adaptation of the SDQ that incorporates 18 additional items, designed to capture a broader range of insomnia symptoms within insomnia disorder. |
| 35 | Sleep Functional Impact Scale [34] | SFIS | Developed for the use in insomnia: the SFIS was developed to evaluate the impact of insomnia disorder on functioning. |
| 36 | Sleep Hygiene Awareness and Practice Scale [35] | SHAPS | Developed for the use in insomnia: the SHAPS was created as a measure of sleep hygiene knowledge and practice among individuals with insomnia disorder. |
| 37 | Sleep Impact Scale [36] | SIS | Developed for the use in insomnia: the SIS was designed to measure the impact of insomnia disorder on quality of life. |
| 38 | Sleep Locus of Control Scale [37] | SLOC | Developed for the use in insomnia: the SLOC is intended to capture sleep locus of control orientation in individuals with insomnia. |
| 39 | Sleep-related Behaviours Questionnaire [38] | SRBQ | Developed for the use in insomnia: the SRBQ was developed as a tool to assess safety behaviours in patients with insomnia. |
| 40 | Short-Form of the Sleep-Related Behavior Questionnaire [39] | SRBQ-SF | Developed for the use in insomnia: the SRBQ is a short version of the SRBQ. |
| 41 | Thought Control Questionnaire-Insomnia revised [40] | TCQI-R | Developed for the use in insomnia: the TCQI-R aims to examine thought control strategies in insomnia disorder. |

*Restless legs (RLS)-specific questionnaires*

|  | **Questionnaire** | **Acronym** | **Reason of exclusion** |
| --- | --- | --- | --- |
| 1 | Analog Restless Legs Syndrome Rating Scale [41] | ARLS | Developed for the use in RLS: the ARLS was developed to evaluate RLS severity, urgency to move, and the degree of symptom relief through movement. |
| 2 | International Restless Legs Syndrome Study Group Rating Scale [42] | IRLSSG | Developed for the use in RLS: the IRLSSG was designed to assess RLS severity, including diagnostic and associated features. |
| 3 | Johns Hopkins Restless Legs Severity Scale | JHRLSS | Developed for the use in RLS: the JHRLSS aims to support the clinical evaluation of RLS severity by focusing on the usual onset time of symptoms. |
| 4 | Kohnen Restless Legs Syndrome–Quality of Life instrument [43] | KRLS-QoL | Developed for the use in RLS: the KRLS-QoL was developed to examine the impact of RLS on quality of life. |
| 5 | Post-Sleep Questionnaire for RLS [43] | PSQ-RLS | Developed for the use in RLS: the PSQ-RLS was created to measure sleep dysfunction in patients with RLS. |
| 6 | Restless Legs Syndrome Quality of Life Instrument [44] | RLS-QLI | Developed for the use in RLS: the RLS-QLI was designed as an instrument to assess health-related quality of life specific to RLS. |
| 7 | Restless Legs Syndrome Quality of Life Questionnaire [45] | RLSQoL | Developed for the use in RLS: the RLSQoL aims to evaluate the impact of RLS across multiple life domains, including daily, social and work life, and emotional wellbeing. |
| 8 | Restless Legs Syndrome-Next Day Impact questionnaire [46] | RLS-NDI | Developed for the use in RLS: the RLS-NDI was constructed to assess the impact of sleep disturbance due to RLS on next-day functioning on several domains. |
| 9 | RLS-6 [43] | RLS-6 | Developed for the use in RLS: the RLS-6 was created to quantify RLS symptom severity during both daytime and nighttime. |
| 10 | RLS‐Self‐care Behaviour questionnaire [47] | RLS‐ScBq | Developed for the use in RLS: the RLS-ScBq was developed to assess self-care activities among patients with RLS. |

*Sleep-related breathing disorder (SRBD) or obstructive sleep apnea (OSA)-specific* *questionnaires*

|  | **Questionnaire** | **Acronym** | **Reason of exclusion (criteria)** |
| --- | --- | --- | --- |
| 1 | Barcelona Sleepiness Index [48] | BSI | Developed for the use in SRBD: the BSI was developed to assess excessive daytime sleepiness in SRBD populations. |
| 2 | Calgary Sleep Apnea Quality of Life Index [49] | SAQLI | Developed for the use in sleep apnea: the SAQLI was created as a sleep apnea-specific tool to measure health-related quality of life. |
| 3 | Fletcher and Luckett Sleep Questionnaire [50] | FLSQ | Developed for the use in OSA: the FLSQ was designed to evaluate OSA symptomatology across domains of day- and nighttime functioning. |
| 4 | Functional Outcomes of Sleep Questionnaire [51] | FOSQ | Developed for the use in disorders of excessive sleepiness and OSA: the FOSQ was developed to evaluate functional status outcomes in patients experiencing excessive sleepiness, amongst others OSA. |
| 5 | 10-item Functional Outcomes of Sleep Questionnaire [52] | FOSQ-10 | Developed for the use in disorders of excessive sleepiness and OSA: the FOSQ-10 is a short version of the FOSQ. |
| 6 | Maugeri Obstructive Sleep Apnea Syndrome [53] | MOSAS | Developed for the use in OSA: the MOSAS was developed to assess to both psychological and physical impact of OSA, as well as adherence to CPAP. |
| 7 | Obstructive Sleep Apnea Patient-Oriented Severity Index [54] | OSAPOSI | Developed for the use in OSA: the OSAPOSI was developed as an OSA-specific questionnaire to evaluate physical, functional and emotional symptoms of OSA. |
| 8 | Patient-Reported Apnea Questionnaire [55] | PRAQ | Developed for the use in OSA: the PRAQ was developed as a PROM to assess quality-of-life domains relevant to OSA. |
| 9 | Patient-reported Longitudinal Assessment Tool for OSA [56] | PLATO | Developed for the use in OSA: the PLATO was developed as a tool to track OSA-related symptoms over time and monitor treatment response. |
| 10 | Quebec Sleep Questionnaire [57] | QSQ | Developed for the use in OSA: the QSQ was designed to evaluate health-related quality of life in individuals with OSA. |
| 11 | Rotterdam Daytime Sleepiness Scale [58] | RSS | Developed for the use in OSA: the RSS was constructed as a disease-specific questionnaire evaluating sleepiness attributable to OSA. |
| 12 | Self-Efficacy Measure for Sleep Apnea [59] | SEMSA | Developed for the use in OSA: the SEMSA was created to examine self-efficacy in OSA by assessing perceived risk, treatment benefit, and willingness to use CPAP. |
| 13 | Sleep-Symptom Questionnaire [60] | SSQ | Developed for the use in OSA: the SSQ was designed to assess OSA symptoms, including the functional impact of sleepiness, sleep-disordered breathing, driving impairment and insomnia. |
| 14 | Snore Outcomes Survey [61] | SOS | Developed for the use in SRBD: the SOS was developed to measure sleep-related quality of life in individuals with SRBD with an emphasis on snoring symptoms. |
| 15 | Symptoms, Tiredness, Alertness, Mood and Psychosocial questionnaire [62] | STAMP | Developed for the use in OSA: the STAMP was created as an OSA-specific instrument aiming to evaluate its impact on health-related quality of life. |
| 16 | Visual Analogue Wellbeing Scale [63] | VAWS | Developed for the use in OSA: the VAWS was designed to assess quality of life in individuals with OSA. |

*Hypersomnia/narcolepsy-specific questionnaires*

|  | **Questionnaire** | **Acronym** | **Reason of exclusion** |
| --- | --- | --- | --- |
| 1 | Hypersomnia Severity Index [64] | HSI | Context: the HIS is a hypersomnia-specific tool intended to assess the severity, distress and impairment of hypersomnia in psychiatric populations. Hypersomnolence disorder was explicitly represented in the study sample and discussed within the development paper, suggesting it can be used in this disorder. The discussion elaborates on the use of the HSI in narcolepsy and hypersomnia disorder. |
| 2 | Hypersomnia-specific Beliefs Scale [65] | HBS | Developed for the use in patients with central disorders of hypersomnia: the HBS was created to evaluate negative hypersomnia-specific beliefs in patients with narcolepsy type 1 and idiopathic hypersomnia. |
| 3 | Idiopathic Hypersomnia Severity Scale [66] | IHSS | Developed for the use in idiopathic hypersomnia: the IHSS is intended to measure hypersomnolence symptoms and their consequences. |
| 4 | Italian Questionnaire on Cataplexy [67] | IQC | Developed for the use in narcolepsy: the IQC was developed to query the frequency of loss of muscle strength in several muscle groups in patients with narcolepsy. |
| 5 | Narcolepsy Severity Scale [68] | NSS | Developed for the use in narcolepsy type 1: the NSS was formulated to quantify the severity and consequences of narcolepsy type 1 symptoms. |
| 6 | Patient-reported outcome measure for central disorders of hypersomnolence [69] | PROM-CDH | Developed for the use in central disorders of hypersomnolence: the PROM-CDH was developed to evaluate quality of life in individuals with narcolepsy of idiopathic hypersomnia. |

*Parasomnia-specific questionnaires*

|  | **Questionnaire** | **Acronym** | **Reason of exclusion** |
| --- | --- | --- | --- |
| 1 | International REM Sleep Behavior Disorder Study Group Symptoms Severity Scale [70] | IRBD-SSS | Developed for the use in RBD: the IRBD-SSS was developed to rate the severity of RBD symptoms. |
| 2 | Night Eating Questionnaire [71] | NEQ | Developed for the use in Night Eating Disorder: the NEQ was developed for the measurement of the severity of NEQ, evaluating both behavioural and psychological symptoms. |
| 3 | Nightmare Distress Questionnaire [72] | NDQ | Context: the NDQ was designed to assess the degree of distress experienced upon awakening from nightmares. The NDQ was developed in a sample experiencing chronic nightmares. The NDQ is a widely used questionnaire in nightmare clinical and research settings and was validated in nightmare disordered populations [73]. |
| 4 | Nightmare Effects Survey [74] | NES | Context: the NES aims to evaluate the psychosocial impact of frequent nightmares. The NES is a widely used questionnaire in nightmare research and can be used in nightmare disorder [75]. |
| 5 | Nightmare Frequency Questionnaire [74] | NFQ | Context: the NFQ was developed to measure nightmare frequency retrospectively. The NFQ was developed in a sample experiencing chronic nightmares. The NFQ is a widely used questionnaire in nightmare clinical and research settings and was validated in nightmare disordered populations [73]. |
| 6 | REM Sleep Behavior Disorder Questionnaire – Hong Kong [76] | RBDQ-HK | Developed for the use in rapid eye movement sleep behavior disorder (RBD): the RBDQ-HK was created to assess RBD symptoms, including both dream-related and behavioral features. |
| 7 | Van Dream Anxiety Scale [77] | VDAS | Context: The VDAS was designed to evaluate nightmare frequency and dream-related anxiety caused by nightmares and was developed in nightmare disorder patients. The development paper elaborates on this disorder. |

[1]	Jansson-Fröjmark M, Harvey AG, Lundh L-G, Norell-Clarke A, Linton SJ. Psychometric Properties of an Insomnia-Specific Measure of Worry: The Anxiety and Preoccupation about Sleep Questionnaire. Cogn Behav Ther 2011;40:65–76. https://doi.org/10.1080/16506073.2010.538432.

[2]	Soldatos CR, Dikeos DG, Paparrigopoulos TJ. Athens Insomnia Scale: validation of an instrument based on ICD-10 criteria. J Psychosom Res 2000;48:555–60. https://doi.org/10.1016/S0022-3999(00)00095-7.

[3]	Carney CE, Harris AL, Falco A, Edinger JD. The relation between insomnia symptoms, mood, and rumination about insomnia symptoms. J Clin Sleep Med 2013;9:567–75. https://doi.org/10.5664/jcsm.2752.

[4]	Morin CM, Stone J, Trinkle D, Mercer J, Remsberg S. Dysfunctional beliefs and attitudes about sleep among older adults with and without insomnia complaints. Psychol Aging 1993;8:463–7. https://doi.org/10.1037/0882-7974.8.3.463.

[5]	Morin CM. Insomnia: Psychological assessment and management. New York: Guilford Press; 1993.

[6]	Morin CM, Vallières A, Ivers H. Dysfunctional Beliefs and Attitudes about Sleep (DBAS): Validation of a Brief Version (DBAS-16). Sleep 2007;30:1547–54. https://doi.org/10.1093/sleep/30.11.1547.

[7]	Edinger JD, Wohlgemuth WK. Psychometric comparisons of the standard and abbreviated DBAS-10 versions of the dysfunctional beliefs and attitudes about sleep questionnaire. Sleep Med 2001;2:493–500. https://doi.org/10.1016/S1389-9457(01)00078-8.

[8]	Kim K, Chung S, Cho E, Choi JM, Lee D, Cho I-K. Reliability and Validity of Dysfunctional Beliefs About Sleep-2 (DBS-2), an Ultra-brief Rating Scale for Assessing Dysfunctional Thoughts About Sleep. Sleep Med Res 2022;13:165–70. https://doi.org/10.17241/smr.2022.01403.

[9]	Gradisar M, Lack L, Richards H, Harris J, Gallasch J, Boundy M, et al. The Flinders Fatigue Scale: preliminary psychometric properties and clinical sensitivity of a new scale for measuring daytime fatigue associated with insomnia. J Clin Sleep Med 2007;3:722–8.

[10]	Harvey KJ, Espie CA. Development and preliminary validation of the Glasgow Content of Thoughts Inventory (GCTI): A new measure for the assessment of pre‐sleep cognitive activity. British Journal of Clinical Psychology 2004;43:409–20. https://doi.org/10.1348/0144665042388900.

[11]	Broomfield NM, Espie CA. Towards a valid, reliable measure of sleep effort. J Sleep Res 2005;14:401–7. https://doi.org/10.1111/j.1365-2869.2005.00481.x.

[12]	Kyle SD, Crawford MR, Morgan K, Spiegelhalder K, Clark AA, Espie CA. The Glasgow Sleep Impact Index (GSII): A novel patient-centred measure for assessing sleep-related quality of life impairment in Insomnia Disorder. Sleep Med 2013;14:493–501. https://doi.org/10.1016/j.sleep.2012.10.023.

[13]	Leger D, Scheuermaier K, Raffray T, Metlaine A, Choudat D, Guilleminault C. HD-16: a new quality of life instrument specifically designed for insomnia. Sleep Med 2005;6:191–8. https://doi.org/10.1016/j.sleep.2005.03.013.

[14]	Jansson-Fröjmark M, Harvey AG, Flink IK. Psychometric properties of the Insomnia Catastrophizing Scale (ICS) in a large community sample. Cogn Behav Ther 2020;49:120–36. https://doi.org/10.1080/16506073.2019.1588362.

[15]	Hudgens S, Phillips-Beyer A, Newton L, Seboek Kinter D, Benes H. Development and Validation of the Insomnia Daytime Symptoms and Impacts Questionnaire (IDSIQ). The Patient - Patient-Centered Outcomes Research 2021;14:249–68. https://doi.org/10.1007/s40271-020-00474-z.

[16]	Ruiz C, Guilera G, Gómez-Benito J. Development of a scale to assess the diurnal impact of insomnia. Psychiatry Res 2011;190:335–41. https://doi.org/10.1016/j.psychres.2011.05.015.

[17]	Bastien C. Validation of the Insomnia Severity Index as an outcome measure for insomnia research. Sleep Med 2001;2:297–307. https://doi.org/10.1016/S1389-9457(00)00065-4.

[18]	Kraepelien M, Blom K, Forsell E, Hentati Isacsson N, Bjurner P, Morin CM, et al. A very brief self-report scale for measuring insomnia severity using two items from the Insomnia Severity Index - development and validation in a clinical population. Sleep Med 2021;81:365–74. https://doi.org/10.1016/j.sleep.2021.03.003.

[19]	Jansson M, Linton SJ. The development of insomnia within the first year: A focus on worry. Br J Health Psychol 2006;11:501–11. https://doi.org/10.1348/135910705X57412.

[20]	Hallit S, Sacre H, Haddad C, Malaeb D, Al Karaki G, Kheir N, et al. Development of the Lebanese insomnia scale (LIS-18): a new scale to assess insomnia in adult patients. BMC Psychiatry 2019;19:421. https://doi.org/10.1186/s12888-019-2406-y.

[21]	Waine J, Broomfield NM, Banham S, Espie CA. Metacognitive beliefs in primary insomnia: Developing and validating the Metacognitions Questionnaire – Insomnia (MCQ-I). J Behav Ther Exp Psychiatry 2009;40:15–23. https://doi.org/10.1016/j.jbtep.2008.03.002.

[22]	Watts FN, Coyle K, East MP. The contribution of worry to insomnia. British Journal of Clinical Psychology 1994;33:211–20. https://doi.org/10.1111/j.2044-8260.1994.tb01115.x.

[23]	David BM. Sleep quality and daytime functioning in primary insomnia: a prospective study. Doctoral thesis. Loughborough University, 2008.

[24]	Moul DE, Germain A, Buysse DJ. Investigating the insomnia severity spectrum using the Pittsburgh Insomnia Rating Scale (PIRS). Sleep Epidemiology 2021;1:100003. https://doi.org/10.1016/j.sleepe.2021.100003.

[25]	Nicassio PM, Mendlowitz DR, Fussell JJ, Petras L. The phenomenology of the pre-sleep state: The development of the pre-sleep arousal scale. Behaviour Research and Therapy 1985;23:263–71. https://doi.org/10.1016/0005-7967(85)90004-X.

[26]	Crönlein T, Langguth B, Popp R, Lukesch H, Pieh C, Hajak G, et al. Regensburg Insomnia Scale (RIS): a new short rating scale for the assessment of psychological symptoms and sleep in insomnia; Study design: development and validation of a new short self-rating scale in a sample of 218 patients suffering from insomnia and 94 healthy controls. Health Qual Life Outcomes 2013;11:65. https://doi.org/10.1186/1477-7525-11-65.

[27]	Drake CL, Hays RD, Morlock R, Wang F, Shikiar R, Frank L, et al. Development and Evaluation of a Measure to Assess Restorative Sleep. Journal of Clinical Sleep Medicine 2014;10:733–41. https://doi.org/10.5664/jcsm.3860.

[28]	Fichten CS, Libman E, Creti L, Amsel R, Tagalakis V, Brender W. Thoughts During Awake Times in Older Good and Poor Sleepers—The Self-Statement Test:60+. Cognit Ther Res 1998;22:1–20. https://doi.org/10.1023/A:1018756618012.

[29]	Semler CN, Harvey AG. Monitoring for Sleep-Related Threat: A Pilot Study of the Sleep Associated Monitoring Index (SAMI). Psychosom Med 2004;66:242–50. https://doi.org/10.1097/01.PSY.0000114870.50968.90.

[30]	El rafihi-Ferreira R, Carmo M, Paulos-Guarnieri L, Pires MLN. The Sleep Acceptance Scale (SAS): Development, psychometric properties, and applications. Behavioral Sleep Medicine 2026;24:84–96. https://doi.org/10.1080/15402002.2025.2555864.

[31]	Kazarian SS, Howe MG, Csapo KG. Development of the sleep behavior self-rating scale. Behav Ther 1979;10:412–7. https://doi.org/10.1016/S0005-7894(79)80029-5.

[32]	Espie CA, Brooks DN, Lindsay WR. An evaluation of tailored psychological treatment of insomnia. J Behav Ther Exp Psychiatry 1989;20:143–53. https://doi.org/10.1016/0005-7916(89)90047-5.

[33]	Coyle K, Watts FN. The factorial structure of sleep dissatisfaction. Behaviour Research and Therapy 1991;29:513–20. https://doi.org/10.1016/0005-7967(91)90001-J.

[34]	Bell C, McLeod LD, Nelson LM, Fehnel SE, Zografos LJ, Bowers B. Development and psychometric evaluation of a new patient-reported outcome instrument measuring the functional impact of insomnia. Quality of Life Research 2011;20:1457–68. https://doi.org/10.1007/s11136-011-9885-8.

[35]	Lacks P, Rotert M. Knowledge and practice of sleep hygiene techniques in insomniacs and good sleepers. Behaviour Research and Therapy 1986;24:365–8. https://doi.org/10.1016/0005-7967(86)90197-X.

[36]	Crawford B, Burgess S, Burrell A, Tellefsen C. Development and validation of the sleep impact scale for insomnia. Value in Health 2007;10:A304–5. https://doi.org/10.1016/S1098-3015(10)65105-4.

[37]	Vincent N, Sande G, Read C, Giannuzzi T. Sleep Locus of Control: Report on a New Scale. Behavioral Sleep Medicine 2004;2:79–93. https://doi.org/10.1207/s15402010bsm0202_1.

[38]	Ree MJ, Harvey AG. Investigating Safety Behaviours in Insomnia: The Development of the Sleep-related Behaviours Questionnaire (SRBQ). Behaviour Change 2004;21:26–36. https://doi.org/10.1375/bech.21.1.26.35971.

[39]	Chung HK, Louie K, Chan WS. Development and evaluation of a Chinese short-form of the Sleep-related Behaviors Questionnaire in Hong Kong Chinese adults using item response theory. J Health Psychol 2024;29:255–65. https://doi.org/10.1177/13591053231195518.

[40]	Ree MJ, Harvey AG, Blake R, Tang NKY, Shawe-Taylor M. Attempts to control unwanted thoughts in the night: development of the thought control questionnaire-insomnia revised (TCQI-R). Behaviour Research and Therapy 2005;43:985–98. https://doi.org/10.1016/j.brat.2004.07.003.

[41]	Shaikh AG, Mehndiratta P, Gonzalez C, Koo BB. Analog Restless Legs Syndrome Rating Scale. Eur Neurol 2013;70:195–200. https://doi.org/10.1159/000351780.

[42]	Walters AS, LeBrocq C, Dhar A, Hening W, Rosen R, Allen RP, et al. Validation of the International Restless Legs Syndrome Study Group rating scale for restless legs syndrome. Sleep Med 2003;4:121–32. https://doi.org/10.1016/S1389-9457(02)00258-7.

[43]	Kohnen R, Martinez-Martin P, Benes H, Trenkwalder C, Högl B, Dunkl E, et al. Validation of the Kohnen Restless Legs Syndrome–Quality of Life instrument. Sleep Med 2016;24:10–7. https://doi.org/10.1016/j.sleep.2016.04.019.

[44]	Atkinson MJ, Allen RP, DuChane J, Murray C, Kushida C, Roth T. Validation of the Restless Legs Syndrome Quality of Life Instrument (RLS-QLI): Findings of a Consortium of National Experts and the RLS Foundation. Quality of Life Research 2004;13:679–93. https://doi.org/10.1023/B:QURE.0000021322.22011.d0.

[45]	Abetz L, Vallow SM, Kirsch J, Allen RP, Washburn T, Earley CJ. Validation of the Restless Legs Syndrome Quality of Life Questionnaire. Value in Health 2005;8:157–67. https://doi.org/10.1111/j.1524-4733.2005.03010.x.

[46]	Lasch KE, Abraham L, Patrick J, Piault EC, Tully SE, Treglia M. Development of a next day functioning measure to assess the impact of sleep disturbance due to restless legs syndrome: The restless legs syndrome-next day impact questionnaire. Sleep Med 2011;12:754–61. https://doi.org/10.1016/j.sleep.2011.03.015.

[47]	Odzakovic E, Sandlund C, Hellström A, Ulander M, Blom K, Jernelöv S, et al. Self‐care behaviours in patients with restless legs syndrome (RLS): development and psychometric testing of the RLS‐Self‐care Behaviour questionnaire. J Sleep Res 2025;34. https://doi.org/10.1111/jsr.14390.

[48]	Guaita M, Salamero M, Vilaseca I, Iranzo A, Montserrat JM, Gaig C, et al. The Barcelona Sleepiness Index: A New Instrument to Assess Excessive Daytime Sleepiness in Sleep Disordered Breathing. Journal of Clinical Sleep Medicine 2015;11:1289–98. https://doi.org/10.5664/jcsm.5188.

[49]	Ward F, Reimer MA. Development of a Disease-specific Health-related Quality of Life Questionnaire for Sleep Apnea. Am J Respir Crit Care Med 1998;158:494–503. https://doi.org/10.1164/ajrccm.158.2.9712036.

[50]	Fletcher EC, Luckett RA. The Effect of Positive Reinforcement on Hourly Compliance in Nasal Continuous Positive Airway Pressure Users with Obstructive Sleep Apnea. American Review of Respiratory Disease 1991;143:936–41. https://doi.org/10.1164/ajrccm/143.5_Pt_1.936.

[51]	Weaver TE, Laizner AM, Evans LK, Maislin G, Chugh DK, Lyon K, et al. An instrument to measure functional status outcomes for disorders of excessive sleepiness. Sleep 1997;20:835–43.

[52]	Chasens ER, Ratcliffe SJ, Weaver TE. Development of the FOSQ-10: A Short Version of the Functional Outcomes of Sleep Questionnaire. Sleep 2009;32:915–9. https://doi.org/10.1093/sleep/32.7.915.

[53]	Moroni L, Neri M, Lucioni AM, Filipponi L, Bertolotti G. A new means of assessing the quality of life of patients with obstructive sleep apnea: The MOSAS questionnaire. Sleep Med 2011;12:959–65. https://doi.org/10.1016/j.sleep.2011.07.010.

[54]	Piccirillo JF, Gates GA, White DL, Schectman KB. Clinical Epidemiology/Outcomes Research. Obstructive Sleep Apnea Treatment Outcomes Pilot Study. Otolaryngology–Head and Neck Surgery 1998;118:833–44. https://doi.org/10.1016/S0194-5998(98)70277-3.

[55]	Abma IL, Rovers M, IJff M, Hol B, Westert GP, van der Wees PJ. The development of a patient-reported outcome measure for patients with obstructive sleep apnea: the Patient-Reported Apnea Questionnaire (PRAQ). J Patient Rep Outcomes 2017;1:14. https://doi.org/10.1186/s41687-017-0021-6.

[56]	Kirsch D, Abbasi-Feinberg F, Davies C, Gamaldo C, Thomas S, Koochaki P, et al. The Patient-reported Longitudinal Assessment Tool for OSA (PLATO): development and validation of a new clinical tool to assess response to obstructive sleep apnea treatment in adults. Journal of Clinical Sleep Medicine 2025;21:1743–58. https://doi.org/10.5664/jcsm.11790.

[57]	Lacasse Y. A new standardised and self-administered quality of life questionnaire specific to obstructive sleep apnoea. Thorax 2004;59:494–9. https://doi.org/10.1136/thx.2003.011205.

[58]	Van Knippenberg FCE, Passchier J, Heysteck D, Shackleton D, Schmitz P, Poublon RML, et al. The Rotterdam Daytime Sleepiness Scale: A New Daytime Sleepiness Scale. Psychol Rep 1995;76:83–7. https://doi.org/10.2466/pr0.1995.76.1.83.

[59]	Weaver TE, Maislin G, Dinges DF, Younger J, Cantor C, McCloskey S, et al. Self-Efficacy in Sleep Apnea: Instrument Development and Patient Perceptions of Obstructive Sleep Apnea Risk, Treatment Benefit, and Volition to Use Continuous Positive Airway Pressure. Sleep 2003;26:727–32. https://doi.org/10.1093/sleep/26.6.727.

[60]	Kump K, Whalen C, Tishler P V, Browner I, Ferrette V, Strohl KP, et al. Assessment of the validity and utility of a sleep-symptom questionnaire. Am J Respir Crit Care Med 1994;150:735–41. https://doi.org/10.1164/ajrccm.150.3.8087345.

[61]	Gliklich RE, Wang P-C. Validation of the Snore Outcomes Survey for Patients With Sleep-Disordered Breathing. Arch Otolaryngol Head Neck Surg 2002;128:819. https://doi.org/10.1001/archotol.128.7.819.

[62]	Mehta N, Mandavia R, Patel A, Zhang H, Liu Z, Kotecha B, et al. Patient‐reported outcome measure for obstructive sleep apnea: Symptoms, Tiredness, Alertness, Mood and Psychosocial questionnaire: Preliminary results. J Sleep Res 2020;29. https://doi.org/10.1111/jsr.12960.

[63]	Masa JF, Jiménez A, Durán J, Carmona C, Monasterio C, Mayos M, et al. Visual analogical well-being scale for sleep apnea patients: validity and responsiveness. Sleep and Breathing 2011;15:549–59. https://doi.org/10.1007/s11325-010-0399-3.

[64]	Kaplan KA, Plante DT, Cook JD, Harvey AG. Development and validation of the Hypersomnia Severity Index (HSI): A measure to assess hypersomnia severity and impairment in psychiatric disorders. Psychiatry Res 2019;281:112547. https://doi.org/10.1016/j.psychres.2019.112547.

[65]	Hazumi M, Ito W, Okubo R, Wada M, Honda M. Development and validation of the hypersomnia-specific beliefs scale. Sleep Med 2020;75:256–62. https://doi.org/10.1016/j.sleep.2020.06.012.

[66]	Dauvilliers Y, Evangelista E, Barateau L, Lopez R, Chenini S, Delbos C, et al. Measurement of symptoms in idiopathic hypersomnia. Neurology 2019;92. https://doi.org/10.1212/WNL.0000000000007264.

[67]	Vignatelli L, D’Alessandro R, Mosconi P, Ferini-Strambi L, Guidolin L, De Vincentiis A, et al. Health-related quality of life in Italian patients with narcolepsy: the SF-36 health survey. Sleep Med 2004;5:467–75. https://doi.org/10.1016/j.sleep.2004.04.003.

[68]	Dauvilliers Y, Beziat S, Pesenti C, Lopez R, Barateau L, Carlander B, et al. Measurement of narcolepsy symptoms: The Narcolepsy Severity Scale. Neurology 2017;88:1358–65. https://doi.org/10.1212/WNL.0000000000003787.

[69]	Bijlenga D, De Boer J, Fronczek R, Tiemensma J, Lammers GJ. Patient-reported outcome measure for central disorders of hypersomnolence (PROM-CDH): development and validation. Sleep Med 2025;133:106589. https://doi.org/10.1016/j.sleep.2025.106589.

[70]	Fantini ML, Postuma RB, Puligheddu M, Rieu I, Venel E, Figorilli M, et al. Validation of the International REM Sleep Behavior Disorder Study Group Symptoms Severity Scale (IRBD-SSS): a new tool to assess RBD clinical severity. J Neurol 2024;271:7264–71. https://doi.org/10.1007/s00415-024-12702-9.

[71]	Martínez MP, Miró E, Arriaza R. Evaluation of the Distress and Effects Caused By Nightmares: A Study of the Psychometric Properties of the Nightmare Distress Questionnaire and the Nightmare Effects Survey . Sleep and Hypnosis 2005;7:29–41.

[72]	Belicki K. The relationship of nightmare frequency to nightmare suffering with implications for treatment and research. Dreaming 1992;2:143–8. https://doi.org/10.1037/h0094355.

[73]	Böckermann M, Gieselmann A, Pietrowsky R. What does nightmare distress mean? Factorial structure and psychometric properties of the Nightmare Distress Questionnaire (NDQ). Dreaming 2014;24:279–89. https://doi.org/10.1037/a0037749.

[74]	Krakow B, Hollifield M, Schrader R, Koss M, Tandberg D, Lauriello J, et al. A controlled study of imagery rehearsal for chronic nightmares in sexual assault survivors with PTSD: A preliminary report. J Trauma Stress 2000;13:589–609. https://doi.org/10.1023/A:1007854015481.

[75]	Schmid C, Kröner-Borowik T, Hansen K, Weßlau C, Steil R. German versions of the Nightmare Effects Survey (NES) and the Nightmare Frequency Questionnaire (NFQ): Psychometric properties in a sample of adult chronic nightmare sufferers. Dreaming 2017;27:232–50. https://doi.org/10.1037/drm0000052.

[76]	Li SX, Wing YK, Lam SP, Zhang J, Yu MWM, Ho CKW, et al. Validation of a new REM sleep behavior disorder questionnaire (RBDQ-HK). Sleep Med 2010;11:43–8. https://doi.org/10.1016/j.sleep.2009.06.008.

[77]	Ağargün MY, Kara H, Bilici M, Çilli AS, Telci M, Semiz ÜB, et al. The Van Dream Anxiety Scale: A subjective measure of dream anxiety in nightmare sufferers. Sleep and Hypnosis 1999;1:204–11.
